# Clinical impact and cost-effectiveness of vaccinating infants and adolescents against invasive meningococcal B disease in the Netherlands

**DOI:** 10.1186/s12916-026-04651-z

**Published:** 2026-02-10

**Authors:** Thomas Otten, Mirjam Knol, Lard Montesano Montessori, Pieter de Boer, Anneke Steens

**Affiliations:** National Institute for Public Health and the Environment, De Bilt, Netherlands

**Keywords:** MenB, Vaccine, Pharmacoeconomics, National immunization program, Modeling, Cost-effectiveness

## Abstract

**Background:**

Invasive meningococcal B disease (IMD-B) causes morbidity and mortality among infants and adolescents in the Netherlands. While multiple vaccines against IMD-B are licensed by the European Medicines Agency, none of them is currently part of the Dutch National Immunization Program (NIP). We evaluated the clinical impact and cost-effectiveness of different IMD-B vaccination strategies for Dutch infants and adolescents.

**Methods:**

We developed a static, single-cohort Markov model to estimate the lifetime number of IMD-B cases and deaths prevented, as well as the incremental cost-effectiveness ratio (ICER), of vaccinating infants with 4CMenB (age 0; 2 + 1 schedule) or adolescents with 4CMenB, MenB-fHBp, or MenABCWY + MenB-fHBp (age 15; 1 + 1 schedule), compared with no IMD-B vaccination. The analysis adopted a societal perspective, including costs and quality-adjusted life years (QALYs) related to vaccination, adverse events, acute IMD-B, long-term sequelae, productivity losses of patients and caregivers, special education needs, and out-of-pocket expenses for patients and their families. We also conducted a threshold analysis for the incidence of IMD-B and a systematic uncertainty assessment.

**Results:**

For infants, the use of 4CMenB would prevent 11.14 IMD-B cases and 0.85 IMD-B-related deaths in one birth cohort of 166,073 infants over a lifetime. For adolescents, IMD-B vaccination would prevent 7.24–8.57 cases and 0.25–0.29 deaths in a single cohort of 197,782 adolescents, depending on which vaccine is used. The ICER was €594,056/QALY for 4CMenB in infants, while for adolescents the ICER ranged between €717,287/QALY and €890,023/QALY, depending on the vaccine type used. These ICERs exceed the commonly used cost-effectiveness thresholds (€20,000 to €80,000/QALY gained) in the Netherlands, rendering vaccination not cost-effective. This outcome proved robust in deterministic and probabilistic sensitivity analyses, as well as in scenario analyses. The threshold analysis demonstrated that IMD-B vaccination may only become cost-effective at a €80,000/QALY threshold with more than a sixfold increase in incidence.

**Conclusions:**

The modelled IMD-B vaccination programs resulted in the prevention of limited morbidity and mortality at a high financial burden. The inclusion of any of the evaluated vaccines in the Dutch NIP for infants or adolescents is not cost-effective in any target group at conventional Dutch cost-effectiveness thresholds given current IMD-B incidence levels.

**Supplementary Information:**

The online version contains supplementary material available at 10.1186/s12916-026-04651-z.

## Background

Invasive meningococcal disease caused by serogroup B (IMD-B) is a life-threatening bacterial infection and an important cause of morbidity and mortality. The overall incidence was 0.6–0.7/100,000 in the Netherlands in recent years (2023–2024) [1], and the disease carries a high case fatality and a high probability of lifelong sequelae [2]. The burden of disease associated with IMD-B is particularly pronounced among infants and adolescents, as the incidence is highest in the age groups 0–5 years and 15–24 years, and the case-fatality rate is highest among individuals aged 0–14 years [3].

Currently, one vaccine, 4CMenB, is licensed by the European Medicines Agency (EMA) for infants [4]. Two vaccines, 4CMenB and MenB-fHBp, are licensed by the EMA for adolescents [4, 5]. Additionally, two pentavalent MenABCWY vaccines have become available [6–8]. None of these vaccines is currently part of the Dutch National Immunization Program for children (NIP). Past considerations by the National Immunization Technical Advisory Group (NITAG) recommended against including IMD-B vaccination in the NIP, due to the low number of IMD-B cases, and the health benefits do not outweigh the individual burden of vaccination, including side effects and the need for multiple doses. Furthermore, the vaccine was considered expensive, with unfavorable cost-effectiveness compared to conventional Dutch thresholds [9].

A cost-effectiveness analysis (CEA) in the Dutch context in 2013 estimated that IMD-B vaccination of infants on a 3 + 1 dosing regimen would only be cost-effective at a €50,000 per quality-adjusted life year (QALY) threshold if disease incidence increased significantly or vaccine prices dropped substantially [10]. Notably, the CEA also provided important insight into the clinical burden potentially prevented by the vaccination program. However, since 2013, new developments have emerged that could impact the outcomes substantially. These include the EMA-approved 2 + 1 schedule for 4CMenB, lowering vaccination costs [11], a slight increase in IMD-B incidence in 2021–2024 [1], new data on vaccine effectiveness (VE) [12–15], and the proportion of IMD-B strains covered per vaccine, affecting VE [13, 16], as well as on the risk of long-term sequelae given IMD-B [2, 17]. Furthermore, no previous studies have included adolescents in their evaluations, despite the relatively high incidence and the availability of new vaccines for this age group, such as the MenB-fHBp (licensed by the EMA in 2017 [18]) and two pentavalent MenABCWY vaccines (licensed by the Food and Drug Administration (FDA) [6, 7]). A pentavalent vaccine could also improve the acceptability of IMD-B vaccination by aligning with the existing MenACWY vaccination in the Dutch NIP for adolescents.

Here, we evaluated the clinical impact and cost-effectiveness of the use of IMD-B vaccination in the Dutch NIP. We compared 4CMenB as a 2 + 1 schedule for infants and 4CMenB, MenB-fHBp, and MenABCWY + MenB-fHBp as 1 + 1 schedules for adolescents with no IMD-B vaccination. The analysis accounts for updated values for IMD-B incidence, sequelae burden, and VE. The preliminary results of this analysis are used to inform the Dutch NITAG regarding the inclusion of IMD-B vaccines in the NIP [3].

## Methods

### Analysis framework

We built a static, single-cohort Markov model with monthly model cycles (1 month as 30.4 days) and a lifetime horizon. The analysis was performed for two cohorts: firstly, infants (age 0), comparing 4CMenB with no vaccination, and, secondly, adolescents (age 15), comparing 4CMenB, MenB-fHBp, and MenABCWY + MenB-fHBp with no vaccination. Cohort sizes were based on the population forecast [19] published in 2023 predicting the population for the year 2025.

The analysis was conducted from a societal perspective. The results incorporate costs and effects (in QALYs) of vaccination, acute IMD-B, complications and sequelae of IMD-B, productivity losses, costs for special education as a result of IMD-B sequelae, and costs that fall onto the patient and their families. We evaluated the vaccines based on clinical outcomes, i.e., the number of IMD-B cases and related deaths avoided and the incremental cost-effectiveness ratio (ICER), i.e., additional costs (€) per additional QALY gained by the vaccination compared to no vaccination. As the abovementioned vaccines do not prevent serogroup B carriage [20–22] and thus do not provide herd protection, only direct effects on clinical outcomes were considered. Health effects and costs were discounted at a rate of 1.5% and 3% per year, respectively [23].

### Model structure

**Fig. 1:**
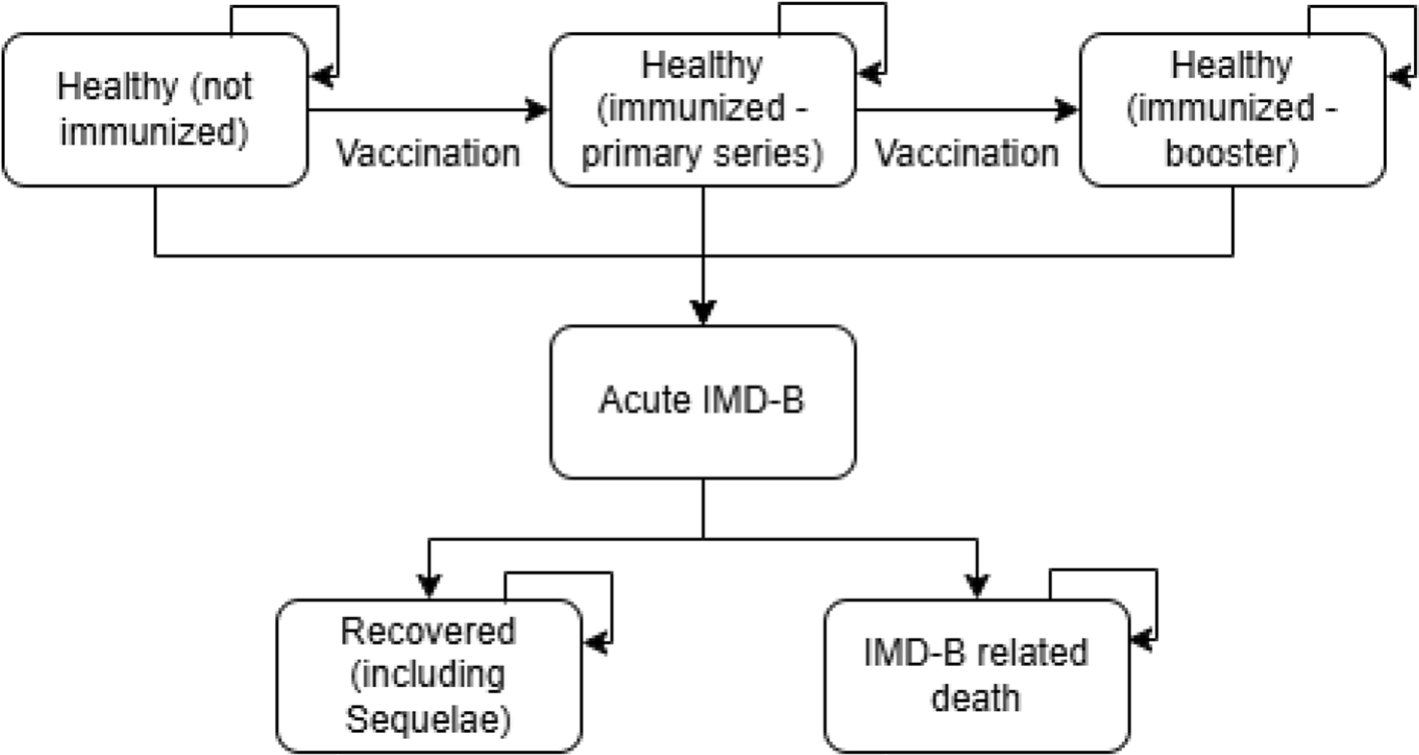
Model structure

We built a model with six health states (Fig. 1): Healthy (not immunized), healthy (immunized — primary series [infants]/first dose [adolescents]), healthy (immunized — booster [infants]/second dose [adolescents]), acute IMD-B, recovered, and death. Individuals in the model transition between health states as follows: All individuals start in the healthy (not immunized) health state. Nonimmunized individuals can remain in this state or transition to the healthy (immunized — primary series) health state if they receive the primary vaccine series. If individuals receive a booster vaccine (infants) or second dose (adolescents), they transition to the healthy (immunized — booster/second dose) health state. If individuals do not contract IMD-B, they remain in a “healthy” health state until death. If an individual contracts IMD-B, they transition to the acute IMD-B health state. From here, individuals can move to the recovered health state, or they can move to the IMD-B-related death health state. The recovered health state includes individuals who developed sequelae after recovering from acute IMD-B. Individuals who recover from IMD-B remain in this state until background mortality applies.

Background mortality (i.e., death not directly caused by IMD-B) applies to all living health states. We used tunnel health states (transition health states to capture time-dependent effects) to account for changes in costs and quality of life after the first year of recovery from IMD-B.

#### Vaccination schedules

We implemented a schedule of two doses for the primary series and one booster dose (2 + 1 schedule) for infants at 2, 4, and 15 months of age, and a 2-dose schedule with a 6-month interval for adolescents aged 15 years; see Table 1.

**Table 1:** Modelled vaccination schedules and coverage

Age group	Vaccine	Schedule	Modelled vaccination age	Coverage	Source of schedule	Source of coverage
Infants	4CMenB	2 + 1	2, 4 months + 15 months	First dose: 0.95 Second dose: 0.95 Third dose: 0.90	[4]	Vaccination coverage report [24] and expert opinion based on pneumococcal vaccination coverage
Adolescents	4CMenB	1 + 1	15 years, 6 months	First dose: 0.90 Second dose: 0.80	[4]	Vaccination coverage report [24] and expert opinion based on MenACWY coverage (for coverage of those starting vaccination) and completion of the HPV vaccination schedule (ratio no. completing versus no. starting vaccination)
	MenB-fHBp	1 + 1	15 years + 6 months	First dose: 0.90 Second dose: 0.80	[18]	
	MenABCWY + MenB-fHBp	1 + 1	15 years + 6 months	First dose: 0.90 Second dose: 0.80	Schedule assumed to be the same as for MenB-fHBp, with MenABCWY replacing the first MenB-fHBp (and MenACWY)	

### Model parameters

#### Transition probabilities

Age-specific incidence of IMD-B and age-specific case-fatality rates were obtained from Dutch surveillance data from the RIVM and the Netherlands Reference Laboratory for Bacterial Meningitis (NRLBM) [3]. The incidence data were collected between May 2022 and October 2024. Data on the case-fatality rate were collected between 2019 and 2024. Within age groups, the incidence was applied evenly throughout each month.

VE against IMD-B was informed by a systematic review conducted for the German NITAG (Ständige Impfkommission — STIKO) [15]. The systematic review identified four articles [12–14, 25] that report on the VE of 4CMenB for infants and one article that reports on the VE of 4CMenB for adolescents [13]. The STIKO review did not consider the theoretical strain coverage.

For infants, the meta-analysis reported a VE for 4CMenB of 81% (95% confidence interval [95% CI], 68–89) after completing the primary series (2 doses) and after completing the full series [15]. We did not attempt to correct the VE of 4CMenB in infants for Dutch strain coverage, given the considerable uncertainty in Dutch strain coverage estimates [3], and thus used a VE of 81% for infants who received two or three vaccine doses. For infants only, we applied a VE of 35% for the first dose based on values reported by Argante [12] and Wang [13].

For adolescents receiving 4CMenB, we applied a VE of 80.1%. For adolescents receiving MenB-fHBp or MenABCWY, we applied a VE of 94.9%. These VE inputs were combined from different sources: The only article included in the meta-analysis [13] reported a VE of 89% (95% *CI* − 0.6% to + 99%) for 4CMenB. This point estimate for VE is, however, higher than the estimated maximum strain coverage for the Netherlands for 4CMenB (82%) [3]. Given the assumption of no (cross-)protection for at least 18% of the IMD strains, this VE would be unrealistic in the Dutch context. For the VE in the adolescent population, we therefore attempted to correct for the strain coverage. The authors of the VE article [13] refer elsewhere [16] to a 90% strain coverage for the studied population. Using this strain coverage, we corrected the VE for covered strains for 4CMenB in adolescents in the study population by dividing the VE by the strain coverage. This resulted in a VE 80.1% for 4CMenB for adolescents. Because the VE of MenB-fHBp and MenABCWY is unknown, we assumed this VE for covered strains for these vaccines to be equal to 4CMenB and multiplied this with the estimated *maximum* strain coverage for the specific vaccine in the Netherlands (i.e., 82% for 4CMenB or 97% for MenB-fHBp/MenABCWY) [3]. This resulted in a VE of 94.9% for MenB-fHBp and MenABCWY. Note that this is an optimistic calculation as the maximum strain coverage for each vaccine is substantially higher than the minimum strain coverage as determined using the 4CMenB antigen sequence type method (strain coverage in adolescents for 4CmenB: 31% and for MenB-fHBp 68%).

Existing data on the decrease in VE over time are scarce and uncertain. Immunogenicity data suggest that the antibody concentrations decline sharply within a year and remain stable afterward [26]. We assumed the duration of protection after the primary series to be 2 years. If infants received a booster or adolescents received a second dose, the duration of protection was assumed to be 5 years after the primary series was completed (or 1 dose in adolescents).

VE was assumed to decrease linearly from the beginning of protection until VE reached 0% when the duration of protection was completed. Pouwels [10] and Christensen [27] have taken similar approaches to model the decrease of VE over time in their CEAs. For both age groups, we modelled VE as starting 1 month after each dose was administered. Table 2* contains the most relevant inputs concerning the transition probabilities applied in the model.*

**Table 2:** Inputs that influence transition probabilities

Type of transition probability	Age group of application (in years)	Input (SD)	Source
Monthly incidence (per 100,000)	< 1	0.503 (0.101)	Dutch surveillance data of RIVM/NRLBM (May 2022–October 2024)
	1–2	0.322 (0.077)	
	2–4	0.165 (0.032)	
	5–14	0.045 (0.009)	
	15–18	0.193 (0.033)	
	19–24	0.132 (0.016)	
	25–64	0.019 (0.003)	
	≥ 65	0.022 (0.005)	
Case-fatality rate	< 5	0.077 (0.024)	Dutch notification data of RIVM (2019–2024)
	5–14	0.079 (0.043)	
	15–24	0.034 (0.015)	
	25–44	0.063 (0.043)	
	45–64	0.022 (0.022)	
	≥ 65	0.070 (0.038)	
VE 4CMenB	Applied in infants (age 0 years)	0.81 (0.053)	[12–15, 25]
	Applied in adolescents (age 15 years)	0.801 (0.089)	[13, 15]
VE MenB-fHBp	Applied in adolescents (age 15 years)	0.949 (0.105)	[13, 15]
VE MenABCWY	Applied in adolescents (age 15 years)	0.949 (0.105)	[13, 15]

*VE* vaccine effectiveness

#### Vaccination costs

We used the list prices of €85.56 per dose for 4CMenB [28] and €80.65 per dose for MenB-fHBp [29]. All vaccine administrations, except for the administration of MenABCWY, were assumed to require their own appointments, i.e., were not part of the existing schedule.

#### Vaccination adverse events

Adverse events costs following administration of 4CMenB in infants were included based on Murdoch et al. (0.1% probability of hospitalization after first vaccination) {Murdoch, 2017, no. 77} and Harcourt et al. (3% probability of GP visit after vaccination) [30].

#### Assumptions regarding the MenABCWY vaccine VE, schedule, and costs

While there is currently no MenABCWY vaccine authorized for use in Europe, we included a hypothetical MenABCWY vaccine for adolescents in our analysis. During the early stages of this CEA, the MenABCWY vaccine that includes the MenB-fHBp component still had marketing authorization [8], so our model is based on the properties of this vaccine.

We thus assumed that the vaccination schedule and effectiveness of MenABCWY would be equivalent to those of MenB-fHBp and modelled MenABCWY as a combination of the MenACWY and MenB-fHBp vaccines.

Currently, MenACWY is included in the NIP for infants and adolescents but does not protect against IMD-B. Adolescents require only one dose of MenACWY, but two doses are needed for the B component. Given this, we assumed MenABCWY would replace the existing single MenACWY dose in the NIP. However, because two doses are needed for full B component coverage, we considered a 1 + 1 schedule, with the MenABCWY vaccine administered as the first dose and MenB-fHBp as the second.

Rather than modeling the separate use of MenACWY, only the incremental costs of MenABCWY were applied. We calculated these as the USA purchasing power parity-adjusted cost of MenABCWY minus the current cost of MenACWY [31], resulting in an incremental cost of €91.10 per dose. Since MenABCWY would replace MenACWY in the schedule, its administration would simply take the place of the existing MenACWY appointment.

#### Acute IMD-B

Individuals who enter the acute IMD-B health state accumulate direct costs and experience a decreased Health-related Quality of Life (QoL). As we did not find QoL inputs for the acute IMD-B health state, we assumed the QoL during this period to be the utility experienced on the ‘worst day of acute IMD-B’ for the duration of the hospitalization [32]. Costs for acute IMD-B were calculated as the sum of the costs of hospitalization, one GP visit, diagnostics, antibiotics, pediatric care (if applicable), and a public health response [33]. For patients who experienced septic shock, additional care was calculated (an additional ICU stay and extra medical assistance for shock).

#### Sequelae

A significant part of patients who recover from severe IMD-B contract long-term sequelae. We included those long-term sequelae that were also included by other published health economic studies on IMD-B [34, 35]. The proportion of patients who experience individual sequelae was based mostly on Viner et al. [36] and Bettinger et al. [37]. In the base case, sequelae were assumed to last for the remaining lifetime of patients. Some sequelae were assumed to decrease in costs and health effect 1 year after recovering from IMD-B. We calculated that the probability of experiencing a sequela was 51%, including a 10% probability of experiencing severe sequelae. Additional file 1 contains all relevant inputs including those used to model sequelae {Viner, 2012 no. 6} {Bettinger, 2013 no. 5} {Zeevat, 2024 no. 2} {Beck, 2021 no. 10} {Ernstsson, 2022 no. 17} {Balieva, 2017 no. 18}.

A recent Dutch study by Middeldorp et al. [2] reported the shares of patients who suffered from severe and mild sequelae in the Netherlands. The authors reported a reduction in the probability of patients suffering from sequelae at 1 year after hospital discharge to 27% of IMD-B cases still experiencing sequelae overall and only 2% of patients still experiencing severe sequelae. We applied these data only in a scenario analysis because the application lacked face validity: Specifically, Middeldorp et al. [2] categorized sequelae solely as “mild” or “severe,” with no differentiation among specific long-term complications. When applied to our base-case analysis, this classification led to limitations: certain severe lifelong sequelae, such as cochlear implants, neurological impairments, motor deficits, and amputations—which typically occur in close to or more than 2% of IMD-B survivors—were disproportionately reduced to below 1% due to the proportional adjustment.

#### Productivity cost

Costs due to loss of productivity were calculated with the friction cost method with a maximum friction period of 85 days in line with Dutch guidelines for health economic evaluation [23]. This method values productivity losses by assuming that workers are replaced (i.e., productivity losses are only valued for the duration that it takes to replace the worker). Age-dependent productivity costs were calculated based on the average hours worked [38], wage per hour [39], and workforce participation [40]. A decrease in productivity was calculated for patients and family members of patients. We calculated a reduction in productivity based on inputs from Zeevat et al. [35].

During hospitalization for acute IMD-B, the patient or one caregiver (if the patient was under 15 years of age) was assumed not to be productive. For patients older than 18 years suffering from sequelae, sequelae-specific productivity losses [35] were multiplied by the friction period. For patients under 18 years of age, one parent per patient with severe sequelae (hearing loss requiring a cochlear implant, severe neurological impairments, mental retardation/low IQ, motor deficits, limb amputation, blindness) and 10% of parents with mild sequelae were assumed to stop working.

#### Special education and institutional care costs

Some patients who experienced sequelae were modelled to receive special education and institutional care based on the methodology and scope used by Zeevat et al. [35]; half of the survivors who suffered from hearing loss, severe neurological disorders, mental retardation/low IQ, motor deficits, limb amputations, blindness, and ADHD required long-term special education. Additional costs for long-term institutional care were assumed for 25% of patients with severe neurological sequelae based on Pouwels et al. [10].

#### Inputs by health state

Table 3 shows the resulting QoL inputs and healthcare costs per individual in each health state per month. All inputs used to calculate health state QoL, and cost inputs can be found in additional file 1.

**Table 3:** Inputs on quality of life (QoL) and healthcare costs per individual in each health state per month

Health states	Quality of life (SD)	Healthcare costs (SD)
Healthy (unvaccinated)	1.000 (0)	€0.00 (0)
Healthy (immunized — primary series)	1.000 (0)	€0.00 (0)
Healthy (immunized — booster)	1.000 (0)	€0.00 (0)
Acute IMD-B	0.688 (0.046)	€10,744.66 (1423.17)
Recovered (1st year)	0.861 (0.019)	€287.51 (776.40)
Recovered (years thereafter)	0.876 (0.018)	€80.15 (206.10)
Death	0.000 (0)	€0.00 (0)

### Cost-effectiveness analysis

The main health economic outcome presented in this analysis is the ICER between the intervention (vaccination) and the comparator (no vaccination). The ICER is interpreted as the additional costs of the intervention to add one QALY compared to the costs and effects of the comparator. In this analysis, for each cohort, the costs and effects of the different vaccinations are compared to the baseline of no vaccination. The ICER is compared to a cost-effectiveness (CE) threshold representing the cost that society is willing to pay for one QALY. If the ICER is higher than the CE-threshold, the intervention is defined as not cost-effective and, from a purely health economic point of view, should not be implemented. In the Netherlands, a CE-threshold of €20,000/QALY is commonly used for preventive measures. However, arguments for a CE-threshold of €50,000/QALY for preventive measures, which is equal to curative care [41], and €80,000/QALY for IMD-B specifically due to its high individual disease burden (9) have been made.

### Threshold analysis

To the best of our knowledge, no published cost-effectiveness analysis has found immunization against IMD-B to be cost-effective at currently observed incidence rates, at the vaccine list price, and under standard recommended assumptions [10, 34, 42]. We conducted a threshold analysis to investigate the incidence at which immunization against IMD-B could become cost-effective. Specifically, we applied a scaling factor to all age-adjusted incidences. The scaling factor represents the multiple by which the current incidence would need to increase for vaccination to become cost-effective at specified cost-effectiveness thresholds. We reported both the scaling factor and the resulting incidence at which vaccination could be cost-effective for infants and adolescents.

### Uncertainty assessment

We used the TRUST tool [43] to identify and communicate uncertainties. To explore input uncertainty, we used the following distributions for the different types of parameters: We conducted standard probabilistic analyses (5000 iterations), deterministic sensitivity analyses, and scenario analyses to investigate the sensitivity of model results to the variation in individual parameters and assumptions. In the probabilistic analyses, we varied multiple inputs by their 95% CI at the same time. In the deterministic analysis, we varied inputs by their 95% CI one by one. For probabilities and utilities, we used beta distributions, and for all resource use and costs, we used gamma distributions. When parameter uncertainty information was not available, the standard error was set at 20% of the parameter input. In the scenario analyses, we changed key methodological assumptions in univariable and multivariable analyses. The rationale and implementation behind each scenario analysis can be found in the additional file 2.

The VE inputs as well as the duration of protection are highly uncertain due to the generally low IMD-B incidence, the scarce data in the literature, and, consequently, our use of combined evidence from differing contexts. Because of the unavailability of the parameter distributions for these inputs, these uncertainties were not explored in the probabilistic analysis. We therefore performed detailed sensitivity analyses to explore the uncertainty around the VE and the duration of protection.

The results of the deterministic sensitivity analysis are not presented in the main manuscript. Details of the uncertainty assessment including the deterministic sensitivity analysis can be found in additional file 2.

### Validation

The model has been internally validated using the TECH-VER validation questionnaire [44] (see additional file 3). We further imitated some analyses conducted by Beck [34] to externally validate our model (see additional file 3). The costs have been externally validated by implementing the costs from the cost of illness study by Zeevat et al. [35] in our model. The results of this can be found in scenario analysis c).

## Results

### Base-case results

According to our modelled results (Table 4), if no IMD-B vaccination is implemented in the NIP, 86.70 infants in the 0-year-old cohort (166,073 individuals) or 62.20 adolescents in the 15-year-old cohort (197,782 individuals) would develop IMD-B during their lifetime. Without vaccination, IMD-B would cause 4.86 or 2.62 deaths in the infant and adolescent cohorts, respectively. The inclusion of 4CMenB in the NIP for infants would reduce the number of IMD-B cases by 11.14 and the number of IMD-B-related deaths by 0.86. The inclusion of any IMD-B vaccine in the NIP for adolescents would reduce the number of cases by 7.24–8.70 and would reduce the number of IMD-B-related deaths by 0.25–0.30 per vaccinated cohort. The results presented in Table 4 demonstrate a modest clinical impact at a high price, which reflects the low incidence of IMD-B.

**Table 4:** Population-level impact of IMD-B vaccination in the Netherlands (mean of probabilistic analysis with 5000 iterations). Note that the costs per person for no vaccination resemble the health care costs for IMD-B

**Population (cohort size)**	**Intervention**	**Total cases**	**Total deaths**	**Total costs** (in million)	**Incremental cases**	**Incremental deaths**	**Incremental costs (in million)**
Infants — age 0 (166,073)	No IMD-B vaccination	86.70	4.86	€4.58	//	//	//
	4CMenB (2 + 1)	75.56	4.00	€58.34	11.14	0.86	− €53.76
Adolescents — age 15 (197,782)	No IMD-B vaccination	62.20	2.62	€2.15	//	//	//
	4CMenB (1 + 1)	54.96	2.37	€40.64	7.24	0.25	− €38.49
	MenB-fHBp (1 + 1)	53.50	2.32	€38.91	8.70	0.30	− €37.76
	MenABCWY + MenB-fHBp (1 + 1)	53.60	2.34	€38.94	8.60	0.28	− €37.79

*QALYs* quality-adjusted life years

Table 5 shows the average lifetime health economic outcomes per cohort of the different IMD-B vaccination schedules. We estimated that vaccinating infants is more cost-effective than vaccinating adolescents. When comparing the different vaccination schedules to no vaccination in the base-case analyses, the results indicated that the ICER of the different scenarios range between €594,056/QALY (4CMenB in infants) and €890,023/QALY (4CMenB in adolescents). The ICERs presented in Table 5 exceed the commonly used threshold values (€20,000–€80,000/QALY) in the Netherlands [23], rendering vaccination not cost-effective. The probabilistic analyses similarly resulted in 0% of iterations being cost-effective even at an €80,000/QALY threshold for any of the vaccine schedules.

**Table 5:** Cost-effectiveness results for IMD-B vaccination per individual in the Netherlands (mean of probabilistic analysis, 5000 iterations). Note that the costs per person for no vaccination resemble the health care costs for IMD-B

Population	Treatment	Cost	QALY	Incremental costs	Incremental QALYs	ICER
Infants (age 0)	No IMD-B vaccination	€27.63	43.89863	//	//	//
	4CMenB (2 + 1)	€351.28	43.89918	€323.64	0.00054	€594,056/QALY
Adolescents (age 15)	No IMD-B vaccination	€10.86	40.36020	//	//	//
	4CMenB (1 + 1)	€205.50	40.36042	€194.64	0.00022	€890,023/QALY
	MenB-fHBp (1 + 1)	€196.72	40.36046	€185.93	0.00026	€719,919/QALY
	MenABCWY + MenB-fHBp (1 + 1)	€196.90	40.36046	€186.06	0.00026	€717,287/QALY

*QALYs* quality-adjusted life years. *ICER* incremental cost-effectiveness ratio

### Threshold analysis

Table 6 presents the results of the threshold analysis, showing that the disease incidence has to increase substantially to render vaccination cost-effective. The population-level incidence of IMD-B is estimated at 0.6 cases per 100,000 population per year. For infants, the incidence would need to increase by a factor ranging from 6.49 to 18.13 for the 4CMenB vaccine to be considered cost-effective. This corresponds to an absolute incidence of 3.89–10.87 IMD-B cases per 100,000 population per year. For adolescents, the required increase in incidence ranges from 8.07 to 24.30 for the MenABCWY vaccine to achieve cost-effectiveness, which translates to an incidence of 4.84–14.57 cases per 100,000 population per year. Additional file 2 presents the results for all four vaccines and per age group.

**Table 6:** Threshold analysis

	**CE-threshold**	**Yearly incidence per 100,000**	**Scaling factor**
Current		0.60	1.00
4CMenB — infants	€20,000/QALY	10.87	18.13
	€80,000/QALY	3.89	6.49
MenABCWY — adolescents	€20,000/QALY	14.57	24.30
	€80,000/QALY	4.84	8.07

CE-threshold, cost-effectiveness threshold

### Uncertainty assessment

**Fig. 2:**
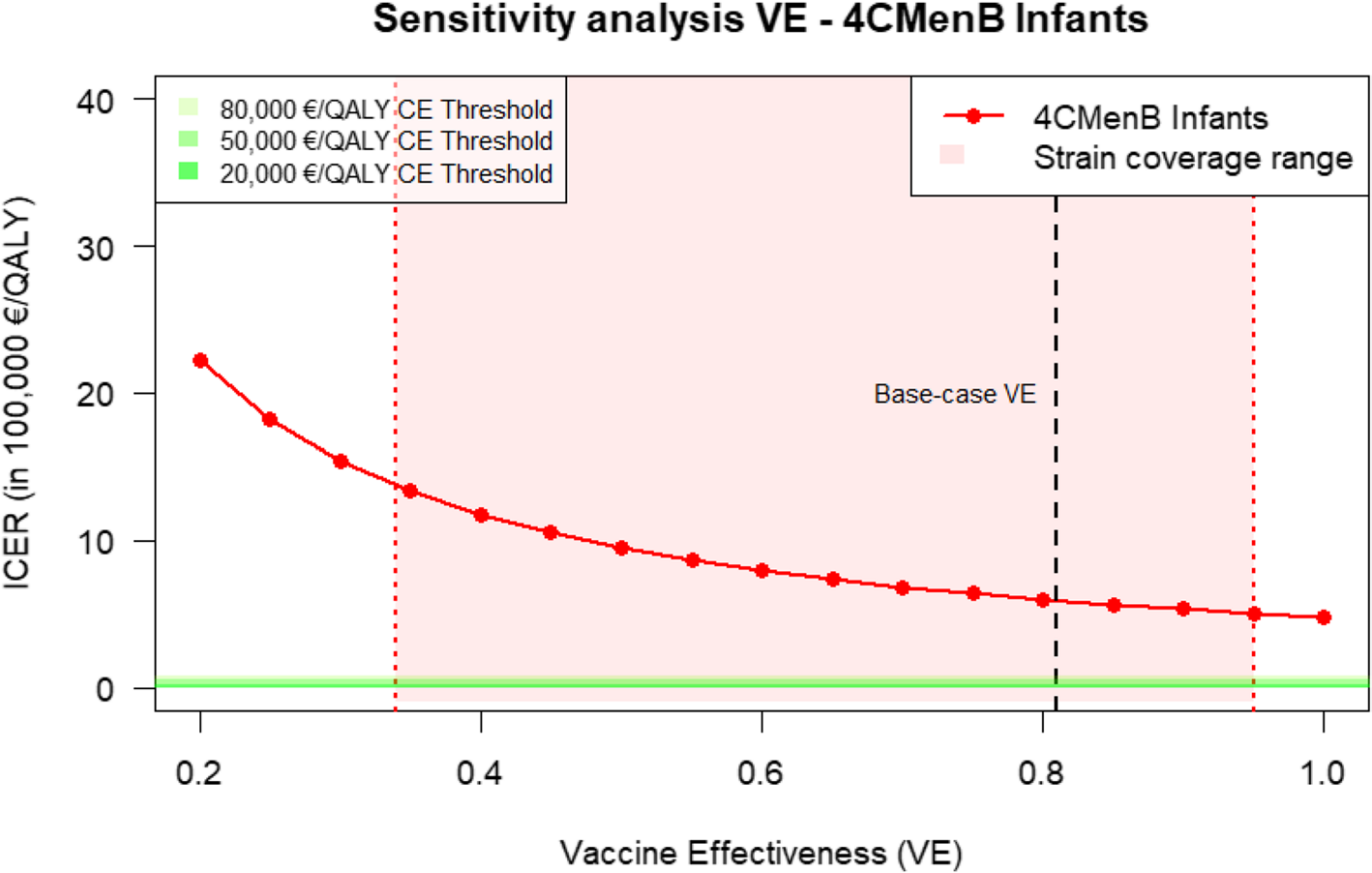
Sensitivity analyses of incremental cost-effectiveness ratio (ICER) to variation in the vaccine effectiveness (VE) for 4CMenB in infants. The red range in the graph shows the range of the Dutch theoretical strain coverage for 4CMenB. The green lines at the bottom indicate different cost-effectiveness thresholds. The black vertical line indicates the base-case VE

**Fig. 3:**
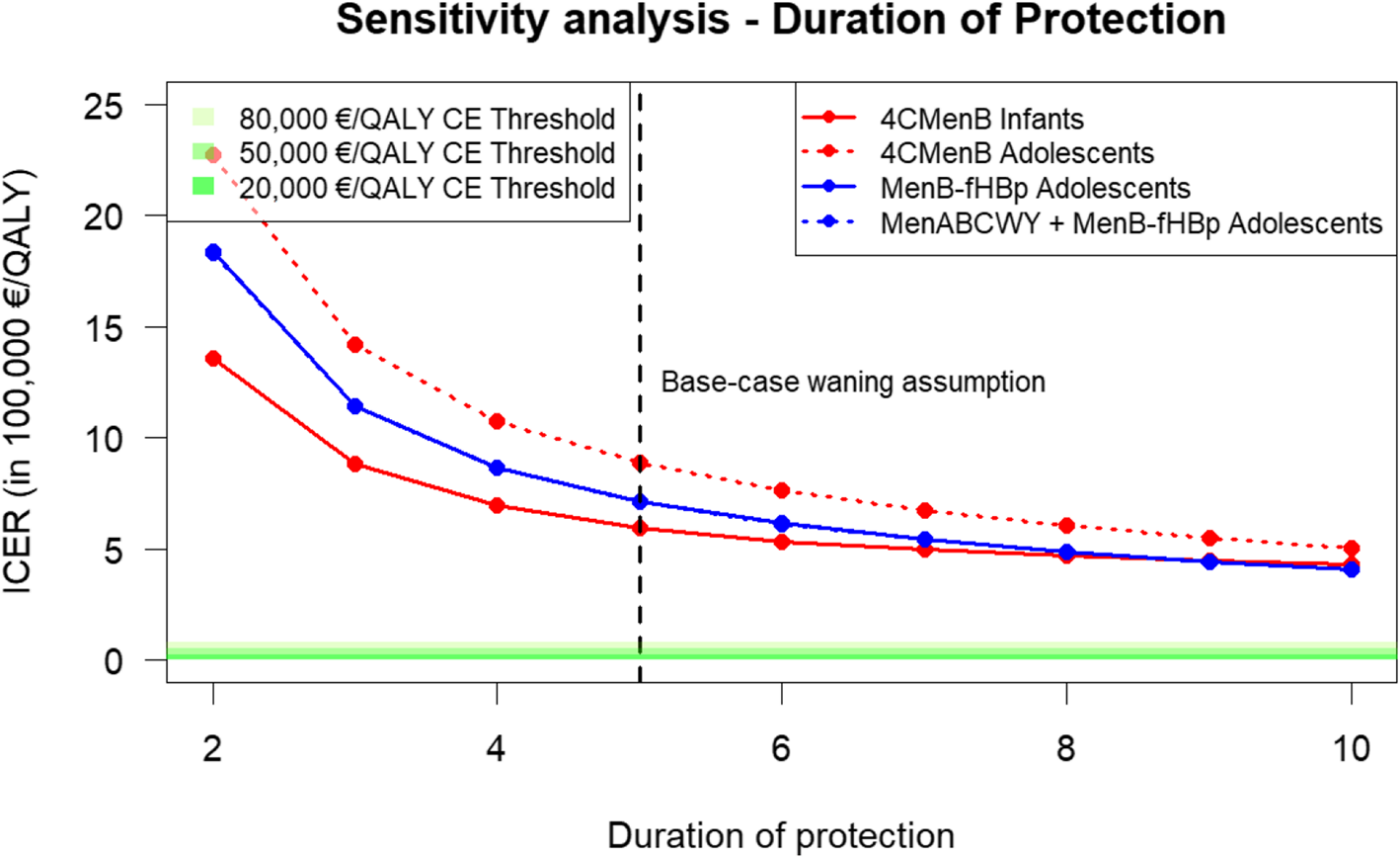
Sensitivity analyses of incremental cost-effectiveness ratio (ICER) to variation in duration of protection using a range of maximum duration of protection between 2 and 10 years. The green lines at the bottom indicate different cost-effectiveness thresholds. The black vertical line indicates the base-case waning input. The lines of the MenB-fHBp and MenABCWY + MenB-fHBp schedules overlap fully as input parameters were similar

The sensitivity analyses indicated that the uncertainty around VE and duration of protection results in a wide range of ICERs (Figs. 2 and 3). In Fig. 2, we only show results for 4CMenB since it is the schedule with the lowest ICER. The results of the remaining analyses can be found in additional file 2. The analysis shows that the results for the uncertainty around the VE for 4CMenB for infants range from €1,334,181/QALY to €505,953/QALY. For the duration of protection, the ICERs range between €1,354,361/QALY and €428,664/QALY for a duration of protection between 2 and 10 years. Both figures illustrate that with increasing VE and duration of protection, the ICER would decrease but remain well above common CE-thresholds.

### Scenario analyses

**Fig. 4:**
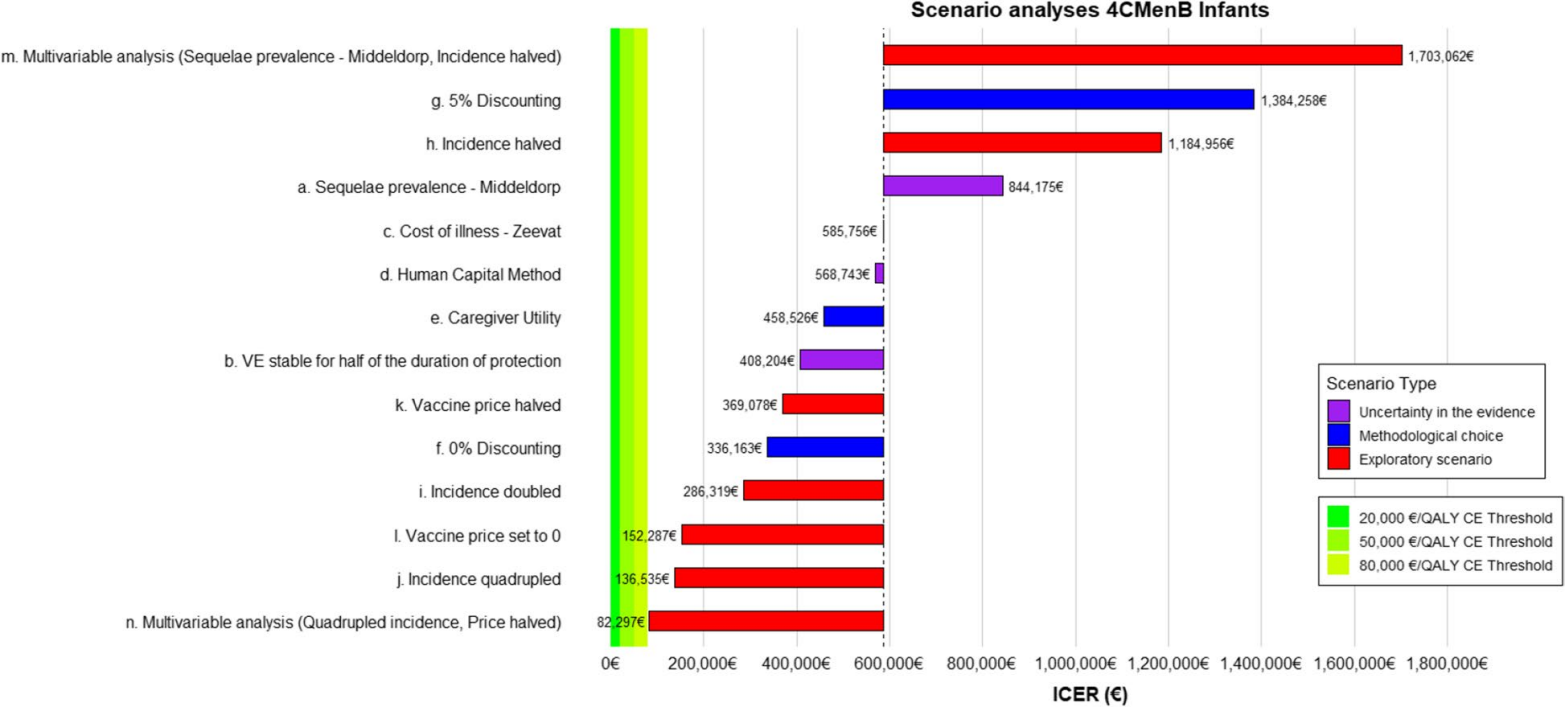
Results of the scenario analyses for 4CMenB in infants: scenario analyses of uncertainty in the evidence (a. applying the sequelae prevalence from Middeldorp [2], b. VE remains stable for half of the duration of protection instead of decreasing from the start, c. applying the cost of illness from Zeevat [35]), scenario analyses of alternative methodological choices (d. applying the human capital method to value productivity, e. including caregiver utility, f. applying 0% discounting rates, g. applying 5% discounting rates), exploratory scenario analyses (h. halving the incidence of IMD-B, i. doubling the incidence of IMD-B, j. quadrupling the incidence of IMD-B, k. halving the price per vaccine dose, l. setting the vaccine price to 0 m), multivariable analysis (sequelae prevalence of Middeldorp, halved incidence), and n. multivariable analysis (quadrupled incidence, price halved)

Figure 4 presents the results of the scenario analyses for infants. The relative increase and direction in which each scenario analysis influenced the ICER were similar between the four vaccine schedules. The analyses showed that applying different costs of illness or the human capital method to value productivity had very little effect on the ICER estimates. The scenario analysis that decreased the ICER most was scenario n. where the incidence was quadrupled at the same time the price was halved. However, even in this scenario analysis, the ICER remained above all CE-thresholds for all vaccines. In scenario analysis a., sequelae prevalence was implemented according to Middeldorp et al. [2]. As expected, this analysis increased the ICER substantially to €844,175/QALY for 4CMenB for infants and to €1,407,473, €1,135,178, and €1,135,792/QALY for 4CMenB, MenB-fHBp, and MenABCWY for adolescents, respectively.

## Discussion

We found that IMD-B vaccination in Dutch infants or adolescents could prevent 7.24–8.57 cases and 0.25–0.29 deaths per cohort. The cost-effectiveness was estimated to be between €594,056/QALY gained and €890,023/QALY gained, which would not be cost-effective according to the common Dutch CE-thresholds ranging from €20,000/QALY to €80,000/QALY gained. These findings were robust across several sensitivity analyses. The probabilistic analysis showed that with the highest CE-threshold of €80,000/QALY [23], all four vaccination schedules had 0% probability of being cost-effective. The threshold analysis showed that for 4CMenB for children to be cost-effective at a €20,000/QALY or €80,000/QALY CE-threshold, the incidence of IMD-B would have to increase 10.87 or 3.89 cases per 100,000 population per year respectively. For MenABCWY, the most cost-effective vaccine in adolescents, the necessary incidence to be cost-effective at a €20,000/QALY or €80,000/QALY CE-threshold would be 1.84 and 14.57.

The conclusion that IMD-B vaccines are currently not cost-effective is in line with the conclusions of several other CEAs [34, 42] including a CEA from the Netherlands from 2013 [10]. Our results demonstrate a less favorable ICER than the only CEA from the Netherlands (ICER for the infant population our model: €594,056/QALY; Pouwels’ [10] model: €243,778/QALY). Most of the difference in model results can be explained by differences in the IMD-B incidence. Despite recent increases, the incidence remains relatively low in the last decade compared to the incidence used by Pouwels [10] (0.6 cases [our article] vs 1.06 cases [Pouwel’s article] per 100,000 person years). It should be noted that an incidence this high has not been observed in the Netherlands in over 20 years [3]. Beck [34] and Scholz [42] combined different additional disease burden categories (i.e., caregiver quality of life, severity modifiers, adjustment of discount rates) to demonstrate that IMD-B could be cost-effective. The inclusion of such additional disease burden categories such as caregiver QoL is encouraged only for a scenario analysis by the Dutch guideline for health economic evaluations [23].

Our findings are directly relevant to the Dutch NITAG. While clinical impact is a necessary criterion for the Dutch NITAG, cost-effectiveness is not a mandatory requirement. Therefore, even though the ICERs for IMD-B vaccination are substantially higher than the commonly used Dutch cost-effectiveness thresholds, the NITAG may still recommend its inclusion in the Dutch NIP. Notably, despite limited cost-effectiveness, several other countries—including Austria, Czech Republic, France, Greece, Ireland, Italy, Lithuania, Luxembourg, Malta, and Portugal—have already implemented IMD-B vaccination in their national programs [45].

This study has several strengths. Firstly, to the best of our knowledge, this is the first CEA to include 4CMenB, MenB-fHBp, and MenABCWY for different age groups on updated schedules within one framework. Next to updated information on epidemiology and vaccination schedule, the CEA updates and expands previous work done for the Dutch context by including multiple, concrete vaccines and vaccination for adolescents. A second strength is that we conducted a comprehensive uncertainty assessment using the TRUST tool [43]. This was particularly important because of the substantial uncertainty in VE and duration of protection. Despite extensive uncertainty analyses, none of the modelled scenarios resulted in the ICER falling within commonly used CE-thresholds. This included a scenario analysis where the vaccine price was set to 0. Even in this case, the ICER remained well above the applied CE thresholds due to the low disease incidence and additional costs (administration costs, patient and family costs, and adverse events related to the vaccine). The results of the uncertainty assessment support the robustness of our conclusions for the current epidemiological context. The results of our threshold analysis may serve as an indicator for future decision-making, in the event of a continued increase in incidence. Our evaluation also has several weaknesses: because limited evidence is available, we had to make strong assumptions in our base-case analysis concerning the VE and duration of protection. With the help of TRUST [43], we identified two uncertainties that could potentially influence cost-effectiveness but were not quantified in our uncertainty assessment: (1) the potential indirect effects of vaccination and (2) the possibility that the probability vary by age group. Based on the analyses conducted by Beck et al. [34], herd protection could decrease the ICER substantially. In contrast to vaccines against MenC and MenW, no evidence of such effects is currently deemed likely in the IMD-B vaccines [20]. A recent commentary [46] highlights potential age dependency of sequelae development as an important uncertainty surrounding meningococcal disease. We expect that this uncertainty could be impactful if, for example, sequelae are more likely or severe in infants who develop IMD-B.

One topic of further research is the prevalence of sequelae and their duration. In our base-case analysis, the proportion of IMD-B cases with sequelae was, similar to other CEAs [10, 34, 42], informed by data sources from outside of the Netherlands [35–37]. When a decreased sequelae proportion was implemented according to data from the Netherlands [2], the ICER increased substantially. To the best of our knowledge, this is the first CEA to include a decreasing proportion of patients suffering sequelae. If this reduction in sequelae is accurate, it suggests that the disease burden of IMD-B—and, consequently, the cost-effectiveness of IMD-B vaccination—may be lower than previously estimated. Similarly, inputs relating to the VE (strain coverage, duration of protection and VE for specific strains) were all highly uncertain. Figures 2 and 3 demonstrate that based on these inputs, the model results can change substantially. Lastly, our threshold and scenario analyses show that the inclusion of IMD-B vaccines in the NIP may be cost-effective with a substantial increase in IMD-B incidence. While the likelihood of clustered cases has historically been low [1] and outbreak sizes have been small as long as antimicrobial prophylaxis was implemented [47, 48], a gradual increase in incidence, changes in disease characteristics, and a larger future outbreak are nevertheless possible. Continued surveillance therefore remains prudent. The results of the threshold analysis may serve as an indicator for future decision-making based on continued surveillance.

Cost-effectiveness is only one of multiple aspects that are taken into account by the Dutch Health Council in its advice on adding a vaccine to a publicly funded national immunization program [49]. There could be valid reasons to introduce a vaccination program that is not cost-effective based on other legitimate reasons, such as a high individual disease burden that is deemed unacceptable by decision-makers.

## Conclusions

Our CEA of 4CMenB in infants and 4CMenB, MenB-fHBp and MenABCWY in adolescents resulted in ICERs ranging between €594,056/QALY and €890,023/QALY. These ICERs are substantially higher than conventional Dutch CE-thresholds in a range of €20,000–€80,000/QALY gained, meaning that including IMD-B vaccination in the Dutch NIP is, under current epidemiological circumstances, not cost-effective. Despite the substantial uncertainty around VE and duration of protection, none of our scenario analyses moved the ICERs into the range of common CE-thresholds. Continued research on major sources of uncertainty such as VE, theoretical strain coverage and sequelae as well as monitoring of natural fluctuations in IMD-B incidence, which could result in vaccination becoming cost-effective in the future, may nevertheless be valuable.

## Supplementary Information

Additional file 1: Inputs. Table 1. Health State Costs. Table 2. Health State Quality of Life. Table 3. Health costs & resource use. Table 4. Sequelae probabilities. Table 5. Sequelae utilities. Table 6. Sequelae costs. Table 7. Productivity costs by age. Table 8. Special education costs. Table 9. Miscellaneous inputs (Vaccine side effects, Caregiver quality of life, travelling costs). Table 10. Inputs for Scenario analyses a) Sequelae probability inputs. Table 11. Inputs for Scenario analysis b) Cost per case of IMD-B.

Additional file 2: Uncertainty Assessment. Page 2–3 – TRUST Tool. Page 4–5 – Detailed explanation of Scenario analyses. Page 6–9 – Results Scenario analyses for all vaccines. Page 10–13 – Tornado Diagrams for all vaccines. Page 14–17 – Sensitivity analyses.

Additional file 3: Validation. Page 1–6 – Cross-validation – Beck. Page 7–17 – TECH-VER checklist.

## Data Availability

All data supporting the findings of this study are available within the paper and its Supplementary Information.

## Abbreviations

CEA: Cost-effectiveness analysis
ICER: Incremental cost-effectiveness threshold
IMD-B: Invasive meningococcal disease serogroup B
NRLBM: Netherlands Reference Laboratory for Bacterial Meningitis
QALYs: Quality-adjusted life-years
QoL: Quality of life
RIVM: Rijksinstituut voor Volksgezondheid en Milieu
STIKO: Ständige Impfkomission
VE: Vaccine effectiveness
