# Supplementary material for: Clinical impact and cost-effectiveness of vaccinating infants and adolescents against invasive meningococcal B disease in the Netherlands
Source: BMC Med. 2026 Feb 10;24:162. doi: 10.1186/s12916-026-04651-z (PMC12990651; doi:10.1186/s12916-026-04651-z)
Supplement: Supplementary file 1 — Additional file 1: Inputs. Table 1. Health State Costs. Table 2. Health State Quality of Life. Table 3. Health costs & resource use. Table 4. Sequelae probabilities. Table 5. Sequelae utilities. Table 6. Sequelae costs. Table 7. Productivity costs by age. Table 8. Special education costs. Table 9. Miscellaneous inputs (Vaccine side effects, Caregiver quality of life, travelling costs). Table 10. Inputs for Scenario analyses a) Sequelae probability inputs. Table 11. Inputs for Scenario analysis b) Cost per case of IMD-B. [file 12916_2026_4651_MOESM1_ESM.docx]

# Appendix 1 - Table of contents

[**Table of contents 1**](#_Toc198200356)

[Health state inputs 2](#_Toc198200357)

[Table 1 Health state costs 2](#_Toc198200358)

[Table 2 Health State Quality of Life inputs 2](#_Toc198200359)

[Healthcare costs & resource use inputs 3](#_Toc198200360)

[Table 3 Healthcare costs & resource use inputs 3](#_Toc198200361)

[Sequelae inputs (Base-case) 4](#_Toc198200362)

[Table 4 Sequelae probabilities 4](#_Toc198200363)

[Table 5 Sequelae utilities 5](#_Toc198200364)

[Table 6 Sequelae costs 6](#_Toc198200365)

[Productivity costs 7](#_Toc198200366)

[Table 7 Productivity costs by age 7](#_Toc198200367)

[Special education costs 8](#_Toc198200368)

[Table 8 Special education costs 8](#_Toc198200369)

[Miscellaneous inputs 9](#_Toc198200370)

[Table 9 Miscellaneous inputs - Vaccine side effects, Caregiver quality of life, travelling costs 9](#_Toc198200371)

[Scenario analyses inputs 10](#_Toc198200372)

[Table 10 Scenario analysis a) Sequelae probabilities inputs 11](#_Toc198200373)

[Table 11 Scenario analysis c) Cost per case of IMD-B 11](#_Toc198200374)

[References 12](#_Toc198200375)

## Health state costs & disutilities

| **Health State costs** | **Mean** | **SD** | **Source** |
| --- | --- | --- | --- |
| Healthy (non-/immunized) | €0 | €0 | Assumed |
| Acute IMD-B | €10,575.48 | €1399.70 | Combined from Healthcare use and Healthcare costs (acute IMD-B) |
| Recovered (1st year) | €286.98 | €65.60 | Combined from probability of sequelae and costs of sequelae (1^st^ year) |
| Recovered (Years thereafter) | €80.10 | €17.47 | Combined from probability of sequelae and costs of sequelae (Years thereafter) |
| Death | €0 | €0 | Assumed |

### Table 1 Health State costs

| **Health State disutilities** | **Mean** | **SD** | **Source** |
| --- | --- | --- | --- |
| Healthy (non-/immunized) | 1 | 0 | Assumed |
| Acute IMD-B | 0.69 | 0.05 | Combined (QoL from Kennedy (1) for the duration of hospitalization) |
| Recovered (1st year) | 0.86 | 0.02 | Combined from probability of sequelae and costs of sequelae (1^st^ year) |
| Recovered (Years thereafter) | 0.88 | 0.02 | Combined from probability of sequelae and costs of sequelae (Years thereafter) |
| Death | 0 | 0 | Assumed |

### Table 2 Health State Quality of Life inputs, all health state inputs are age-adjusted

## Healthcare costs & resource use inputs

| **Vaccine costs** | **Mean** | **SE** | **Source** |
| --- | --- | --- | --- |
| Administration cost - routine | €8.94 | €1.79* | (2) |
| Administration cost - not routine | €19.27 | €3.85* | (2) |
| 4CMenB - cost per dose | €85.56 | €0.00** | (3) |
| MenB-fHBp - cost per dose | €80.65 | €0.00** | (4) |
| MenABCWY - cost per dose | €91.10 | €0.00** | Combination (5, 6) |
| **Healthcare use (Acute IMD-B)** | | | |
| Duration Hospitalization without Septic Shock | 10.1 days | 2.02 | (2) |
| Duration Hospitalitzation with Septic Shock | 9 days | 0.82 | (2) |
| Duration ICU with Septic Shock | 4 days | 0.61 | (2) |
| Probability septic shock | 17% | 0.01 | (7) |
| **Healthcare costs (Acute IMD-B)** | | | |
| Standard care hospitalization day | €644.00 | €128.80* | (8) |
| Hospitalization day at intensive care unit | €2727.00 | €545.40* | (8) |
| GP visit | €30.87 | €6.17* | (8) |
| Diagnostics | €378.03 | €45.00* | (8) |
| Full course of antibiotics | €2,394.21 | €45.00* | (8) |
| Extra medical assistance with shock | €159.61 | €285.00* | (8) |
| Pediatrician follow-up after recovery | €95.00 | €19.00* | (8) |
| Public health response to a case | €50.00 | €10.00* | (9) |
| Additional immediate hospitalization costs associated with treatment of scars | €1,200.07 | €240.01* | (2) |
| Additional immediate hospitalization costs associated with treatment of amputations | €4,800.26 | €960.05* | (2) |
| * Uncertainty information unavailable, SE assumed to be equal to 20% of the mean  ** Assumed fixed  All costs have been inflated to 2024 values | | | |

### Table 3 Healthcare costs & resource use inputs

## Sequelae inputs (Base-case)

| **Sequelae probabilities** | **Mean** | **SE** | **Source** |
| --- | --- | --- | --- |
| Hearing loss unilateral | 5.64% | 0.018 | (10) |
| Hearing loss bilateral | 3.80% | 0.015 | (10) |
| Hearing loss requiring Cochlear implant | 2.45% | 0.010 | (10) |
| Severe Neurological | 3.58% | 0.009 | (11) |
| Mental retardation/low IQ | 0.52% | 0.007 | (10) |
| Speech problems | 3.56% | 0.014 | (10) |
| Motor deficits | 1.53% | 0.015 | (12) |
| Limb amputation | 1.26% | 0.007 | (10) |
| Epilepsy/seizures | 1.78% | 0.010 | (10) |
| Skin scarring | 6.39% | 0.012 | (11) |
| Renal disease | 2.05% | 0.007 | (11) |
| Blindness | 0.42% | 0.004 | (10) |
| ADHD | 9.66% | 0.027 | (10) |
| Anxiety | 2.18% | 0.014 | (10) |
| Separation anxiety | 5.97% | 0.022 | (10) |

### Table 4 Sequelae probabilities

| **Severe Sequelae disutitilities - First year** | **Mean** | **SE** | **Source** |
| --- | --- | --- | --- |
| Hearing loss unilateral | 0.10 | 0.08 | (2) |
| Hearing loss bilateral | 0.41 | 0.11 | (2) |
| Hearing loss requiring Cochlear implant | 0.41 | 0.11 | (2) |
| Severe Neurological | 0.38 | 0.02 | (2) |
| Mental retardation/low IQ | 0.22 | 0.08 | (13) |
| Speech problems | 0.17 | 0.02 | (13) |
| Motor deficits | 0.44 | 0.30 | (13) |
| Limb amputation | 0.17 | 0.02 | (14) |
| Epilepsy/seizures | 0.36 | 0.04 | (13) |
| Skin scarring | 0.18 | 0.04 | (15) |
| Renal disease | 0.74 | 0.04 | (13) |
| Blindness | 0.25 | 0.06 | (13) |
| ADHD | 0.25 | 0.02 | (13) |
| Anxiety | 0.25 | 0.02 | (13) |
| **Severe Sequelae disutitilities – Years thereafter** | | | |
| Hearing loss unilateral | 0.10 | 0.08 | (2) |
| Hearing loss bilateral | 0.41 | 0.11 | (2) |
| Hearing loss requiring Cochlear implant | 0.45 | 0.11 | (2) |
| Severe Neurological | 0.32 | 0.02 | (2) |
| Mental retardation/low IQ | 0.38 | 0.02 | (13) |
| Speech problems | 0.22 | 0.08 | (13) |
| Motor deficits | 0.17 | 0.02 | (13) |
| Limb amputation | 0.44 | 0.30 | (14) |
| Epilepsy/seizures | 0.17 | 0.02 | (13) |
| Skin scarring | 0.36 | 0.04 | (15) |
| Renal disease | 0.18 | 0.04 | (13) |
| Blindness | 0.74 | 0.04 | (13) |
| ADHD | 0.25 | 0.06 | (13) |
| Anxiety | 0.25 | 0.02 | (13) |

### Table 5 Sequelae utilities

| **Sequelae costs - First year** | **Mean** | **SE** | **Source** |
| --- | --- | --- | --- |
| Hearing loss unilateral | €1,026.98 | 104.90 | (12) |
| Hearing loss bilateral | €3,073.66 | 313.64 | (12) |
| Hearing loss requiring Cochlear implant | €47,785.09 | 4876.07 | (12) |
| Severe Neurological | €3,738.24 | 713.65 | (12) |
| Mental retardation/low IQ | €8,723.60 | 890.07 | (12) |
| Speech problems | €4,263.67 | 434.97 | (12) |
| Motor deficits | €2,530.58 | 258.28 | (12) |
| Limb amputation | €77,332.76 | 4187.58 | (12) |
| Epilepsy/seizures | €694.69 | 70.73 | (12) |
| Skin scarring | €1,355.11 | 307.82 | (12) |
| Renal disease | €0.00 | 0.00 | (12) |
| Blindness | €2,855.60 | 1068.35 | (12) |
| ADHD | €5,309.34 | 541.73 | (12) |
| Anxiety | €78.92 | 45.83 | (12) |
| Hearing loss unilateral | €78.92 | 8.21 | (12) |
| **Sequelae costs – Subsequent years** | | | |
| Hearing loss unilateral | €163.03 | 16.69 | (12) |
| Hearing loss bilateral | €233.64 | 23.84 | (12) |
| Hearing loss requiring Cochlear implant | €3,711.24 | 1228.62 | (12) |
| Severe Neurological | €591.89 | 238.15 | (12) |
| Mental retardation/low IQ | €8,723.60 | 890.07 | (12) |
| Speech problems | €0.00 | 0.00 | (12) |
| Motor deficits | €1,489.07 | 147.55 | (12) |
| Limb amputation | €1,7320.51 | 1767.70 | (12) |
| Epilepsy/seizures | €0.00 | 0.00 | (12) |
| Skin scarring | €0.00 | 0.00 | (12) |
| Renal disease | €146.00 | 15.05 | (12) |
| Blindness | €2,855.60 | 1068.35 | (12) |
| ADHD | €5,309.34 | 541.73 | (12) |
| Anxiety | €78.92 | 45.83 | (12) |
| Hearing loss unilateral | €78.92 | 8.21 | (12) |
| All costs have been inflated to 2024 values | | | |

### Table 6 Sequelae costs

## Productivity costs

| **Productivity cost per age category** | **Mean** | **SD** | Sources |
| --- | --- | --- | --- |
| 15-19 | €189.03 | 67.16 | (16-18) |
| 20-24 | €1,285.73 | 452.55 | (16-18) |
| 25-29 | €2,589.67 | 911.25 | (16-18) |
| 30-34 | €3,151.00 | 1120.98 | (16-18) |
| 35-39 | €3,408.41 | 1212.58 | (16-18) |
| 40-44 | €3,638.42 | 1288.31 | (16-18) |
| 45-49 | €3,684.42 | 1302.75 | (16-18) |
| 50-54 | €3,798.74 | 1355.56 | (16-18) |
| 55-69 | €3,168.61 | 1108.37 | (16-18) |
| 60-64 | €3,061.36 | 1090.98 | (16-18) |
| 65-74 | €557.73 | 192.82 | (16-18) |
| >75 | €373.48 | 131.77 | (16-18) |
| All costs have been inflated to 2024 values | | | |

### Table 7 Productivity costs by age

## Special education costs

| **Special education costs** | **Mean** | **SE** | **Source** | **Applied to** |
| --- | --- | --- | --- | --- |
| Special education costs Cat1.1 | €20,456.05 | 4091.21* | (12) | Motor deficits |
| Special education costs Cat1.2 | €17,854.46 | 3570.89* | (12) |  |
| Special education costs Cat1.3 | €23,038.40 | 4607.68* | (12) |  |
| Special education costs Cat2.1 | €26,701.99 | 5340.40* | (12) | Amputation, Low IQ |
| Special education costs Cat2.2 | €26,702.64 | 5340.53* | (12) |  |
| Special education costs Cat2.3 | €33,050.78 | 6610.16* | (12) |  |
| Special education costs Cat3.1 | €36,757.29 | 7351.46* | (12) | Neurological deficits |
| Special education costs Cat3.2 | €36,648.54 | 7329.71* | (12) |  |
| Special education costs Cat3.3 | €38,564.86 | 7712.97* | (12) |  |
| Special education costs Hearing Loss Blindness | €1,557.60 | 311.52* | (12) | Hearing loss, blindness |
| Special education costs Hearing Loss Blindness VSO | €3,115.20 | 623.04* | (12) |  |
| Special education costs ADHD | €2,804.72 | 560.94* | (12) | ADHD |
| * Uncertainty information unavailable, SE assumed to be equal to 20% of the mean  All costs have been inflated to 2024 values | | | | |

### Table 8 Special education costs

## Miscellaneous inputs

|  | **Mean** | **SE** | **Source** |
| --- | --- | --- | --- |
| **Vaccine Side Effects** | | | |
| Hospitalizations due to vaccination | 0.1% | 0.00005 | (19) |
| GP appointments due to vaccination | 3.02% | 0.00013 | (20) |
| **Travelling costs** | | | |
| Traveling cost hospital | €5.65 | 1.13* | (2) |
| Traveling cost GP | €1.27 | 0.25* | (2) |
| Traveling cost municipal healthcare center | €11.92 | 2.38* | (2) |
| * Uncertainty information unavailable, SE assumed to be equal to 20% of the mean  All costs have been inflated to 2024 values | | | |

### Table 9 Miscellaneous inputs - Vaccine side effects, Caregiver quality of life, travelling costs

## Scenario analyses inputs

| **Sequelae probabilities (First year)** | **Mean** |  |
| --- | --- | --- |
| Hearing loss unilateral | 8.25% | (21) |
| Hearing loss bilateral | 3.85% |  |
| Hearing loss requiring Cochlear implant | 2.83% |  |
| Severe Neurological | 1.20% |  |
| Mental retardation/low IQ | 0.97% |  |
| Speech problems | 5.83% |  |
| Motor deficits | 0.00% |  |
| Limb amputation | 1.01% |  |
| Epilepsy/seizures | 3.64% |  |
| Skin scarring | 7.04% |  |
| Renal disease | 3.55% |  |
| Blindness | 0.09% |  |
| ADHD | 9.91% |  |
| Anxiety | 1.46% |  |
| Separation anxiety | 11.13% |  |
| **Sequelae probabilities (Years thereafter)** | | |
| Hearing loss unilateral | 3.03% | (21) |
| Hearing loss bilateral | 2.04% |  |
| Hearing loss requiring Cochlear implant | 1.01% |  |
| Severe Neurological | 0.50% |  |
| Mental retardation/low IQ | 0.21% |  |
| Speech problems | 1.91% |  |
| Motor deficits | 0.00% |  |
| Limb amputation | 0.52% |  |
| Epilepsy/seizures | 0.96% |  |
| Skin scarring | 3.43% |  |
| Renal disease | 1.10% |  |
| Blindness | 0.17% |  |
| ADHD | 5.18% |  |
| Anxiety | 1.17% |  |
| Separation anxiety | 3.20% |  |
| No probabilistic analysis was conducted | | |

### Table 10 Scenario analysis a) Sequelae probabilities inputs (Inputs adjusted based on Middeldorp [20])

| **Caregiver quality of life** | | | |
| --- | --- | --- | --- |
| Family & network factor | 0.48 | 0.08* | (13) |
| Bereavement factor | 0.09 | 0.05* | (13) |
| Caregiver utility decrement | 0.11 | 0.05* | (13) |
| Caregiving probability | 7.00% | 0.03* | (13) |

### Table 11 Scenario analysis c) including caregiver utility (based on methods by Beck [12])

|  | **Mean** | **Source** |
| --- | --- | --- |
| Cost per case of IMD-B | €41,746.79 | (12) |
| Costs were inflated to 2024 values  No probabilistic analysis was conducted | | |

### Table 12 Scenario analysis c) Cost per case of IMD-B

## References

1. Kennedy ITR, van Hoek AJ, Ribeiro S, Christensen H, Edmunds WJ, Ramsay ME, et al. Short-term changes in the health state of children with group B meningococcal disease: A prospective, national cohort study. PLoS One. 2017;12(5):e0177082.

2. Pouwels KB, Hak E, van der Ende A, Christensen H, van den Dobbelsteen GP, Postma MJ. Cost-effectiveness of vaccination against meningococcal B among Dutch infants: Crucial impact of changes in incidence. Hum Vaccin Immunother. 2013;9(5):1129–38.

3. Medicijnkosten.nl. BEXSERO INJSUSP WWSP 0,5ML: Medicijnkosten.nl; [Available from: <https://www.medicijnkosten.nl/medicijn?artikel=BEXSERO+INJSUSP+WWSP+0%2C5ML+%2B+TOEBEH&id=91325ce387bed983e3ff773cf64f1c68>.

4. Medicijnkosten.nl. TRUMENBA INJSUSP WWSP 0,5ML: Medicijnkosten.nl; [Available from: https<www.medicijnkosten.nlmedicijnartikel=TRUMENBA+INJSUSP+WWSP+0%2C5ML&id=318184922342945170718b7688ccdf34>.

5. Medicijnkosten.nl. NIMENRIX INJPDR FLACON + SOLVENS IN WWSP: Medicijnkosten.nl; [Available from: <https://www.medicijnkosten.nl/medicijn?artikel=NIMENRIX+INJPDR+FLACON+%2B+SOLVENS+IN+WWSP&id=6263e1ce844c79b5df81967601811473>.

6. CDC. Current CDC Vaccine Price List | VFC Program | CDC.

7. Stoof SP, Rodenburg GD, Knol MJ, Rumke LW, Bovenkerk S, Berbers GA, et al. Disease Burden of Invasive Meningococcal Disease in the Netherlands Between June 1999 and June 2011: A Subjective Role for Serogroup and Clonal Complex. Clin Infect Dis. 2015;61(8):1281–92.

8. ZIN. Guideline for Economic Evaluations in Healthcare. National Health Care Institute; 2024.

9. Welte R, van den Dobbelsteen G, Bos JM, de Melker H, van Alphen L, Spanjaard L, et al. Economic evaluation of meningococcal serogroup C conjugate vaccination programmes in The Netherlands and its impact on decision-making. Vaccine. 2004;23(4):470–9.

10. Viner RM, Booy R, Johnson H, Edmunds WJ, Hudson L, Bedford H, et al. Outcomes of invasive meningococcal serogroup B disease in children and adolescents (MOSAIC): a case-control study. Lancet Neurol. 2012;11(9):774–83.

11. Bettinger JA, Scheifele DW, Le Saux N, Halperin SA, Vaudry W, Tsang R, et al. The disease burden of invasive meningococcal serogroup B disease in Canada. Pediatr Infect Dis J. 2013;32(1):e20–5.

12. Zeevat F, Simons JJM, Westra TA, Wilschut JC, van Sorge NM, Boersma C, et al. Cost of Illness Analysis of Invasive Meningococcal Disease Caused by Neisseria Meningitidis Serogroup B in the Netherlands-a Holistic Approach. Infect Dis Ther. 2024;13(3):481–99.

13. Beck E, Klint J, Neine M, Garcia S, Meszaros K. Cost-Effectiveness of 4CMenB Infant Vaccination in England: A Comprehensive Valuation Considering the Broad Impact of Serogroup B Invasive Meningococcal Disease. Value Health. 2021;24(1):91–104.

14. Ernstsson O, Hagberg K, Janssen MF, Bonsel GJ, Korkmaz S, Zethraeus N, et al. Health-related quality of life in patients with lower limb amputation – an assessment of the measurement properties of EQ-5D-3L and EQ-5D-5L using data from the Swedish Amputation and Prosthetics Registry. Disability and Rehabilitation. 2022;44(26):8471–9.

15. Balieva F, Kupfer J, Lien L, Gieler U, Finlay AY, Tomás-Aragonés L, et al. The burden of common skin diseases assessed with the EQ5D™: a European multicentre study in 13 countries. British Journal of Dermatology. 2017;176(5):1170–8.

16. CBS. Dashboard Arbeidsmarkt - Arbeidsparticipatie naar leeftijd en geslacht: Centraal Bureau voor de Statistiek; 2023 [updated 2025–02–19. Available from: <https://www.cbs.nl/nl-nl/visualisaties/dashboard-arbeidsmarkt/werkenden/arbeidsparticipatie-naar-leeftijd-en-geslacht>.

17. Statista. Average monthly wage in the Netherlands by age: Statista; 2022 [updated 2025–02–19. Available from: <https://www.statista.com/statistics/538025/average-monthly-wage-in-the-netherlands-by-age/>.

18. Statista. Average annual working hours in the Netherlands by age: Statista; 2022 [updated 2025–02–19. Available from: <https://www.statista.com/statistics/538657/average-annual-working-hours-in-the-netherlands-by-age/>.

19. Murdoch H, Wallace L, Bishop J, Robertson C, Claire Cameron J. Risk of hospitalisation with fever following MenB vaccination: self-controlled case series analysis. Arch Dis Child. 2017;102(10):894–8.

20. Harcourt S, Morbey RA, Bates C, Carter H, Ladhani SN, de Lusignan S, et al. Estimating primary care attendance rates for fever in infants after meningococcal B vaccination in England using national syndromic surveillance data. Vaccine. 2018;36(4):565–71.

21. Middeldorp M, Steens A, Lagerweij G, van Sorge NM, Freudenburg-de Graaf W, E AMS, et al. The burden of invasive meningococcal disease in the Netherlands, 2011-2020. Vaccine. 2023;41(16):2664–70.
