# Supplementary material for: Clinical impact and cost-effectiveness of vaccinating infants and adolescents against invasive meningococcal B disease in the Netherlands
Source: BMC Med. 2026 Feb 10;24:162. doi: 10.1186/s12916-026-04651-z (PMC12990651; doi:10.1186/s12916-026-04651-z)
Supplement: Supplementary file 2 — Additional file 2: Uncertainty Assessment. Page 2–3 – TRUST Tool. Page 4–5 – Detailed explanation of Scenario analyses. Page 6–9 – Results Scenario analyses for all vaccines. Page 10–13 – Tornado Diagrams for all vaccines. Page 14–17 – Sensitivity analyses. [file 12916_2026_4651_MOESM2_ESM.docx]

Appendix 2 - Analyses

Contents

[Appendix 2 - Analyses 1](#_Toc215143390)

[TRUST Tool 2](#_Toc215143391)

[Explanation of scenario analyses 4](#_Toc215143392)

[Scenario analyses 6](#_Toc215143393)

[Deterministic sensitivity analyses 10](#_Toc215143394)

[Sensitivity analyses 14](#_Toc215143395)

[Sensitivity analyses – Overall VE 14](#_Toc215143396)

[Sensitivity analysis – Duration of protection 17](#_Toc215143397)

## TRUST Tool

|  | | **Sources of uncertainty** | | | | |
| --- | --- | --- | --- | --- | --- | --- |
|  |  | **Transparency** | **Methods** | **Imprecision** | **Bias & Indirectness** | **Unavailability** |
| **Aspects of the model** | **Context/Scope** | // | // |  | // | // |
|  | **Model structure** | // | a)Assumption: Vaccination has no effect on transmission |  | // |  |
|  | **Selection of evidence** | // | b)Using maximum strain coverage inputs to calculate adolescent VE  c) Different sources of evidence for sequelae (UK evidence compared to NL evidence)  d) Evidence was not identified systematically |  | l) Use of UK data as a source of evidence for the prevalence of sequelae | // |
|  | **Inputs** | Not all inputs are currently shown in the Appendix | e) Different sources offer different manners to calculate productivity loss and special education  f)Differences in the inclusion of sequelae between sources  g) Differences in the way sequelae are counted between sources (per sequelae vs per severity)  h) Under-reporting of IMD-B | j)Standard imprecision around inputs  k)The evidence used for adolescents was classified as highly uncertain by the STIKO | m)Calculation of vaccine effectiveness for adolescents is very indirect  n)Calculation of utility during severe disease | o) VE evidence for the Netherlands missing  p)Duration of protection of vaccines  q)Uncertainty information was missing for a number of inputs  r)Sequelae prevalence may be age-dependent |
|  | **Implementation** | // | i) Implementation of utilities connected to sequelae (The model assumes that that disutilities are additive) |  |  |  |
|  | **Outcomes** | // |  |  |  |  |

Table 1 Identified uncertainties

|  | | **Impact on cost-effectiveness** | | |
| --- | --- | --- | --- | --- |
|  |  | **Uncertainty not reflected in PSA?** | **Uncertainty not explored in scenario analysis?** | **High impact on cost-effectiveness?** |
| **Aspects of the model** | **Context/Scope** | // | // | // |
|  | **Model structure** | a) | a) | a) Yes, an effect on transmission could have an effect on the broader population and make the vaccines more cost-effective |
|  | **Selection of evidence** | b) c) d) | d) | d) No, as inputs were recently identified in other articles |
|  | **Inputs** | e) f) g) h) l) m) n) o) p) r) | e) h) n) r) | e) No, productivity loss and special education were not very influential  h) No, severe cases were likely reported  n) No, utility during severe disease is only very short term  r) Yes, as sequelae were impactful, if sequelae are much more likely in infants this may influence cost-effectiveness |
|  | **Implementation** | i) |  |  |
|  | **Outcomes** | // | // | // |

Table 2 Uncertainties not included in current analyses

## Explanation of scenario analyses

| **Type** | **Number** | **Name** | **Implementation** | **Rationale** |
| --- | --- | --- | --- | --- |
| **Uncertainty in the evidence** | a. | Sequelae prevalence - Middeldorp | Adapting the overall sequelae prevalence to that reported for the Netherlands by Middeldorp [33] | In the base-case analyses sequelae probability following IMD-B is informed through data from data from the UK[24]. Middeldorp [33]reports on Dutch data according to which sequelae prevalence decreases within one year. Middeldorp classifies sequelae into severe and mild . We classified the sequelae from the UK study similarly and weighted those according to the probability of severe and mild sequelae. This analysis adapts the share of patients with sequelae to the data reported by Middeldorp. |
|  | b. | VE stable for half of the duration of protection | Keeping the VE at the full VE for half of the duration of protection | Little information is available on the decrease of VE over time in IMD-B vaccines. In our base-case analysis we assume that VE starts decreasing immediately. This analysis investigates the impact of a longer duration with full VE. |
|  | c. | Cost of Illness – Zeevat | Implementing cost per case from Zeevat [23] instead of our own cost analysis | Zeevat [23] conducted a cost-of-illness study. This analysis investigates the impact of using the cost per case calculated by Zeevat. This scenario analysis serves to validate our own cost analysis and investigate whether using different methods drastically changes the results. |
|  | d. | Human Capital Method | Valuing productivity costs with the Human Capital Method instead of the Friction cost method | In line with the Dutch Health Economic guidelines [29]  our analyses use the Friction Cost Method to calculate productivity loss. This analysis investigates the impact of valuing productivity with the Human Capital Method. The Human Capital Method values productivity losses by assuming that productivity losses by assuming that workers do not get replaced (i.e., productivity losses are permanent). |
| **Methodological choice** | e. | Caregiver Utility | Implementing caregiver utility | The Dutch Health Economic guidelines [29] recommend to explore the impact of caregiver utility in scenario analyses. These analyses implement caregiver utility by using inputs and methods from  Beck[10] and Scholz.  Caregivers received a 0.48 family and network factor where the family and network received a penalty which was proportionally to the Quality of Life penalty of patients. For every IMD-related patient death, we counted a permanent 0.09 utility decrement after patient death. Lastly, for every informal caregiver (see productivity costs), we calculated a 0.11 utility penalty. All caregiver QoL was influenced by age and mortality. We assumed an average caregiver age of 29.9 at the birth of the child. |
|  | f. | 0% Discounting | Removing discount factors | Because these are preventive vaccines, their benefits often appear much later in the model. This is true for IMD-B as well, as some sequelae may be life-long. We therefore explore the impact of removing the discount rates for costs and effects. |
|  | g. | 5% Discounting | Increasing discounting for costs and effects to 5% | In this scenario analysis we explore the impact of increasing the discounting rates for costs and effects to a higher level. |
| **Exploratory scenario** | h. | Double incidence | The incidence is doubled | The base-case results indicate that IMD-B vaccines are currently very expensive for the effect that it delivers. This scenario analysis investigates the impact of a substantial increase in the incidence. The analysis is interesting because in the past, the incidence has been higher[2]. |
|  | i. | Halve the price | The price of the vaccine is halved. | The base-case results indicate that IMD-B vaccines are currently very expensive. This scenario analysis investigates the impact of a substantial reduction in the price. |

Table 3 Explanation of scenario analyses

## Scenario analyses


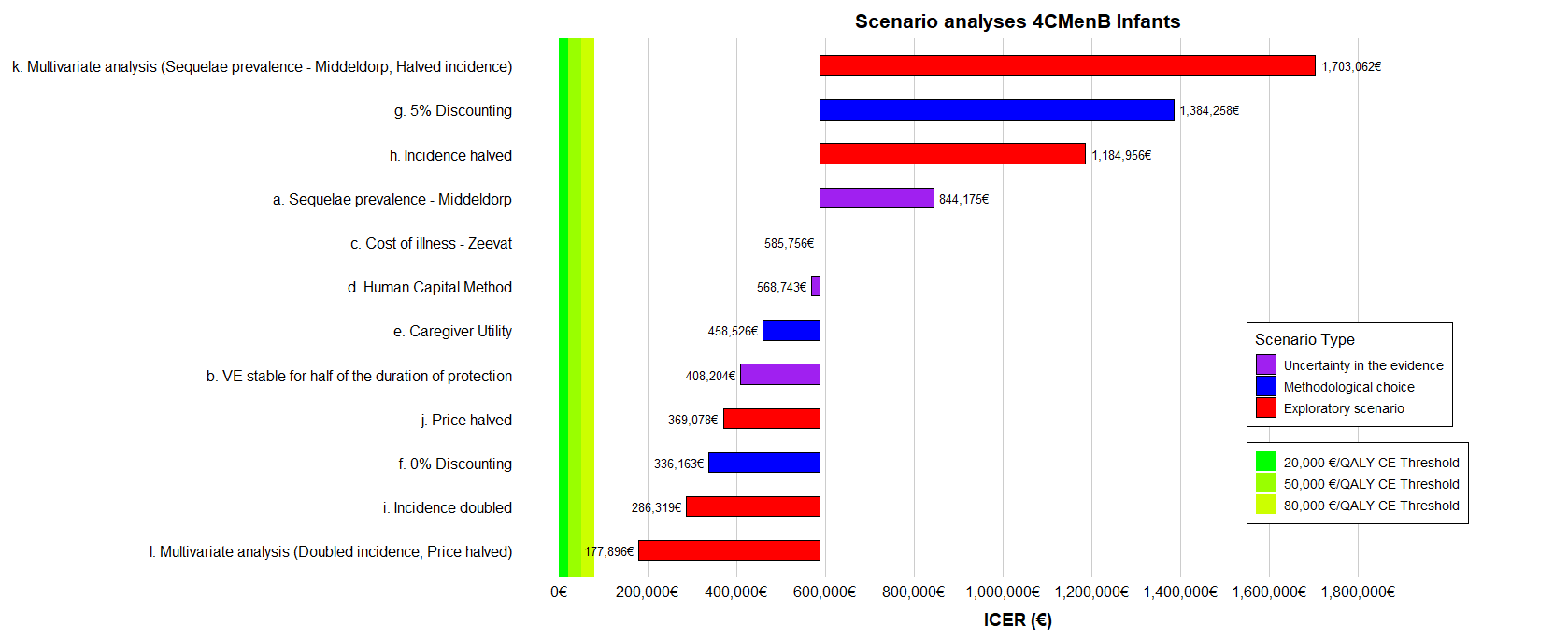


Figure 1 Results of the Scenario analyses for the 4CmenB Infant schedule


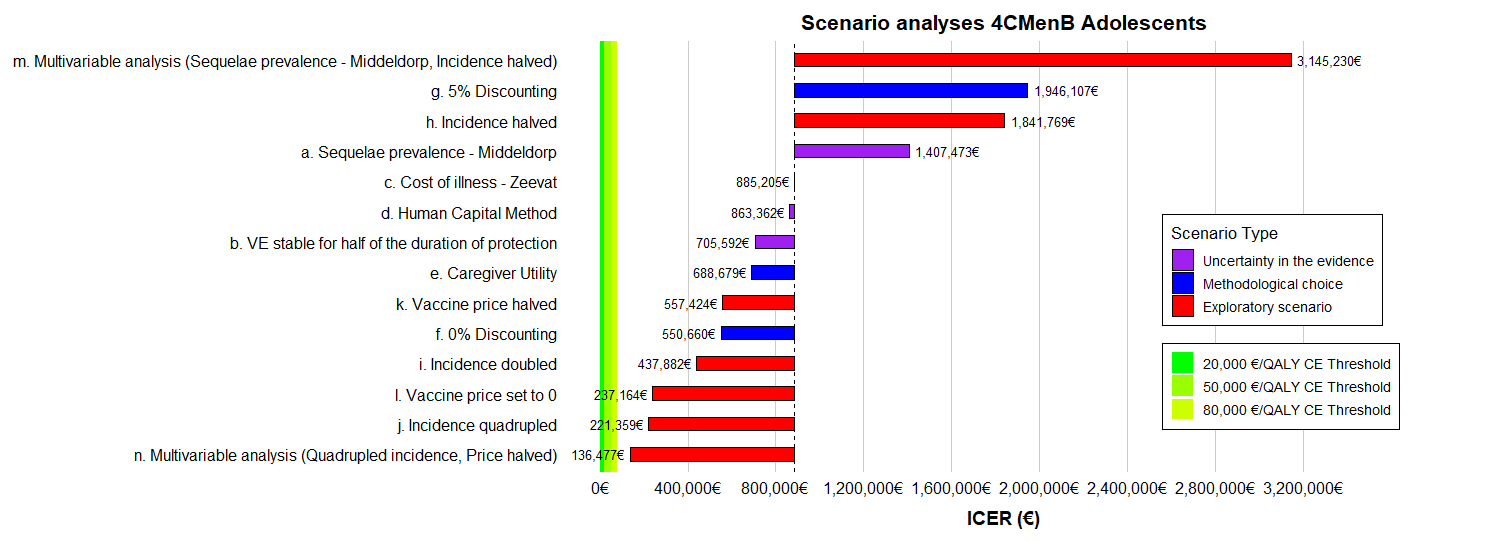


Figure 2 Results of the Scenario analyses for the 4CmenB Adolescent schedule


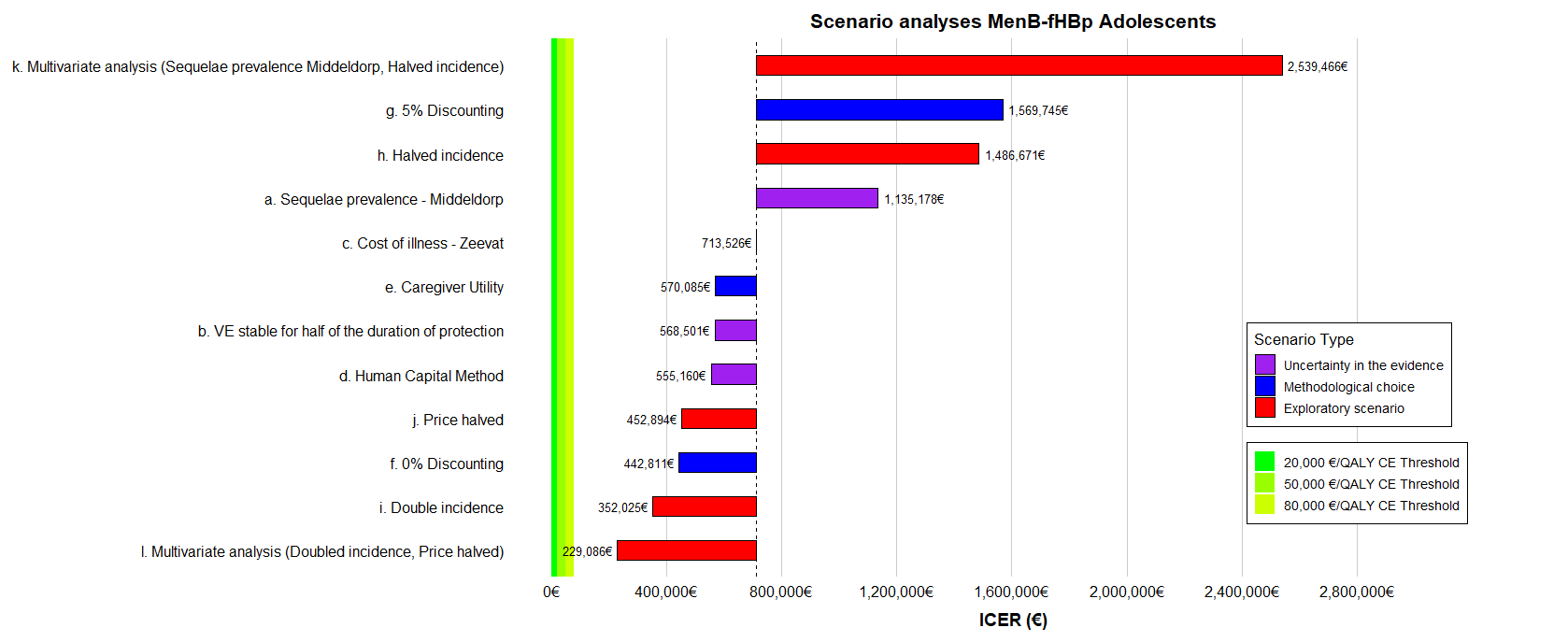


Figure 3 Results of the Scenario analyses for the MenB-fHBp adolescent schedule


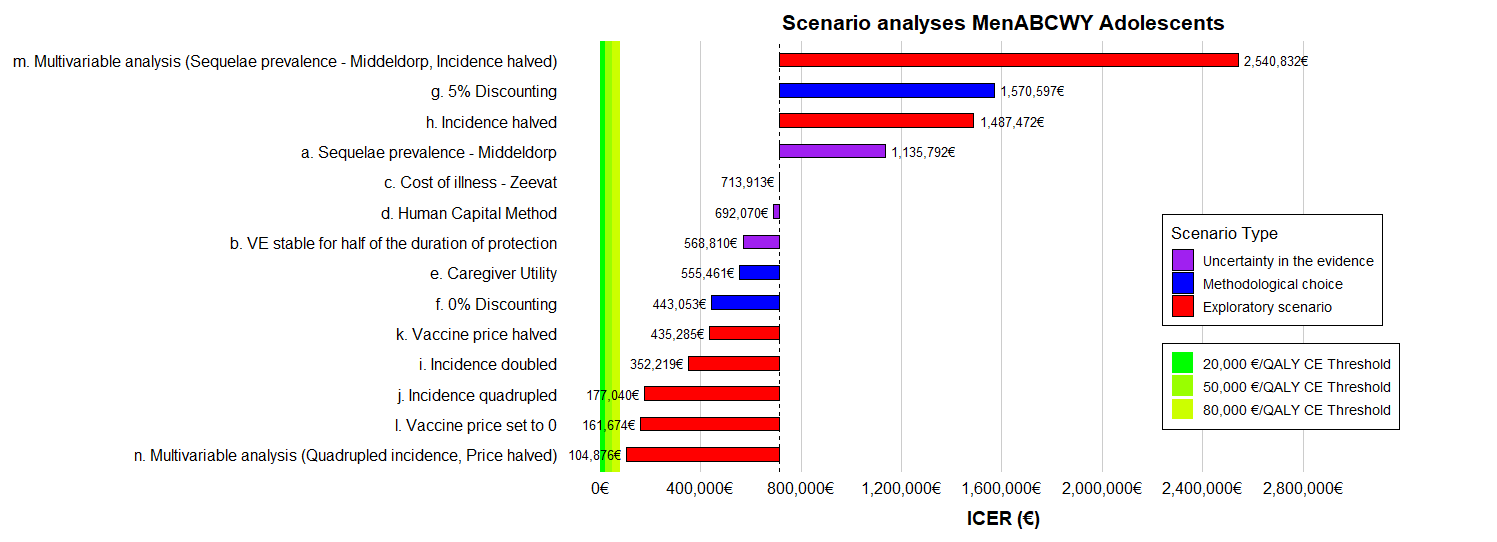


Figure 4 Results of the Scenario analyses for the MenABCWY adolescent schedule

## Deterministic sensitivity analyses

Figure 5 Tornado Diagrams 4CMenB infant schedule

Figure 6 Tornado Diagrams 4CMenB adolescent schedule

Figure 7 Tornado Diagrams MenB-fHBp adolescent schedule

Figure 8 Tornado Diagrams MenABCWY + MenB-fHBp adolescent schedule

## Sensitivity analyses

### Sensitivity analyses – Overall VE


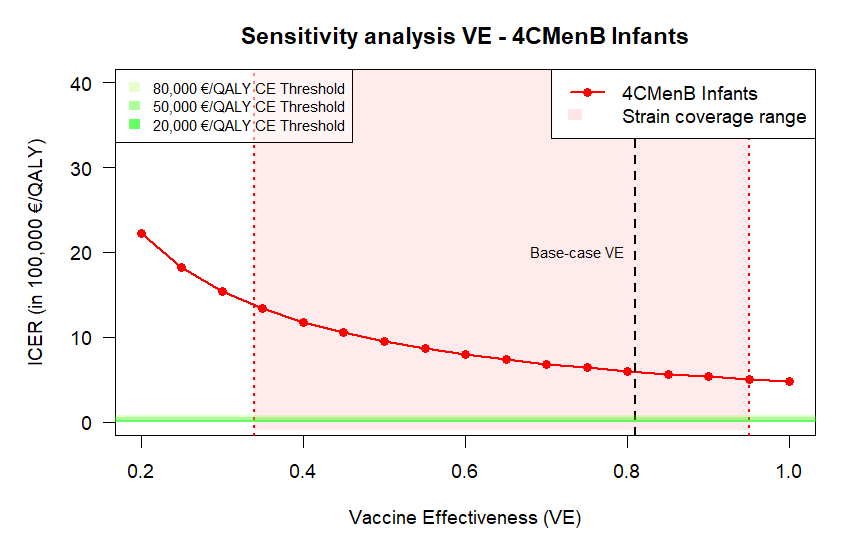


Figure 9 Sensitivity analysis ICER by Vaccine Effectiveness - 4CMenB Infants schedule


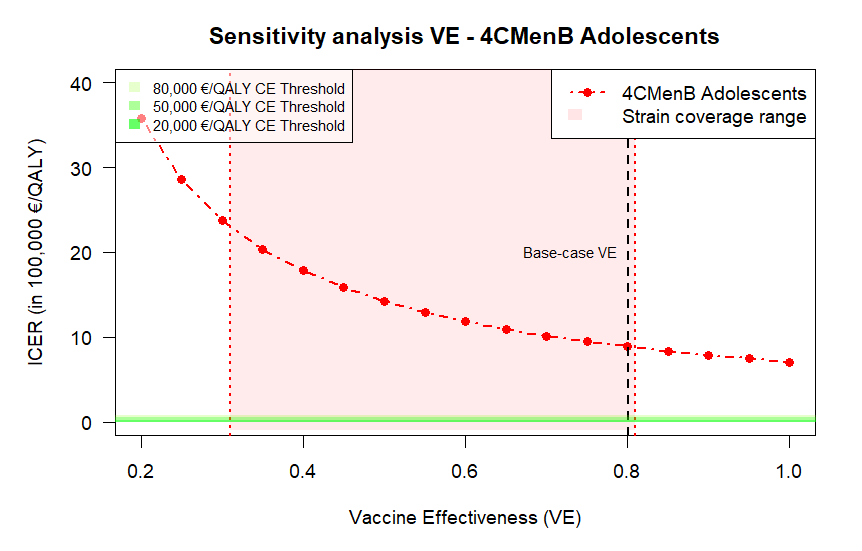


Figure 10 Sensitivity analysis ICER by Vaccine Effectiveness - 4CMenB adolescent schedule


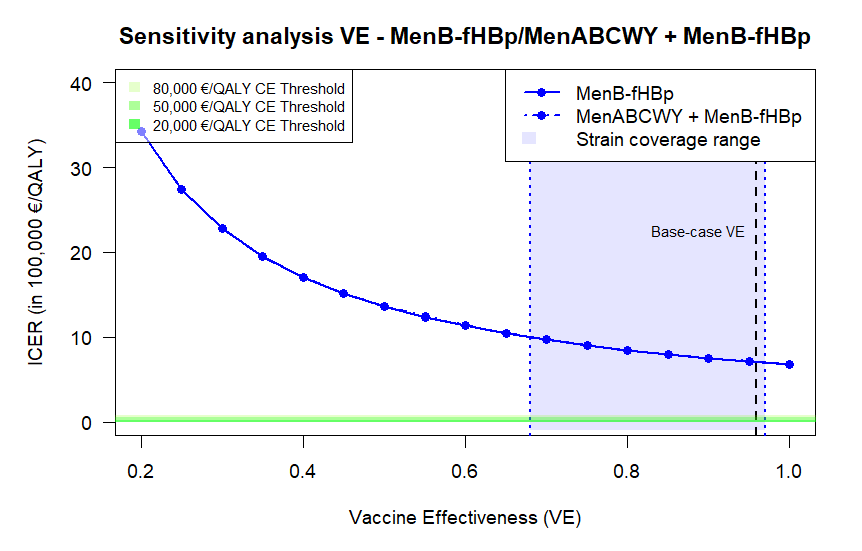


Figure 11 ICER by Vaccine Effectiveness – MenABCWY / MenB-fHBp – Adolescents – **note that “MenB-fHBp” and “MenABCWY + MenB-fHBp” results are so close that the lines overlap completely**

### Sensitivity analysis – Duration of protection

**
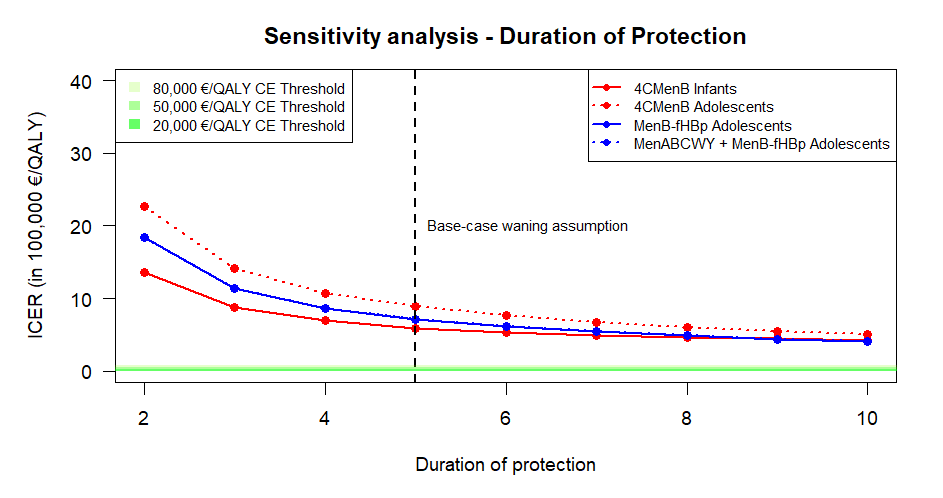
**

Figure 12 ICER by duration of protection – all vaccines – **note that “MenB-fHBp” and “MenABCWY + MenB-fHBp” results are so close that the lines overlap completely**
