# Supplementary material for: Clinical impact and cost-effectiveness of vaccinating infants and adolescents against invasive meningococcal B disease in the Netherlands
Source: BMC Med. 2026 Feb 10;24:162. doi: 10.1186/s12916-026-04651-z (PMC12990651; doi:10.1186/s12916-026-04651-z)
Supplement: Supplementary file 3 — Additional file 3: Validation. Page 1–6 – Cross-validation – Beck. Page 7–17 – TECH-VER checklist. [file 12916_2026_4651_MOESM3_ESM.docx]

# Cross validation - Beck

Beck et al.[1] evaluated the cost-effectiveness of 4CMenB in infants, highlighting in their introduction that previous studies on this topic have generally found 4CMenB not to be cost-effective or only cost-effective at very low prices. The authors argue that the broader effects of MenB vaccination, warrant a more holistic assessment than is often being conducted. Their study includes two analyses: (1) a baseline cost-effectiveness evaluation of 4CMenB and (2) an expanded assessment incorporating five additional disease burden categories (DBCs) to evaluate the vaccine's value more comprehensively. The inclusion of these additional categories results in a favorable ICER for 4CMenB in infants. This stands in contrast to other evaluations that rely on the vaccine’s list price[2, 3], the baseline calculation in the same study, and our analysis.

We think the different conclusions can largely be explained by two factors: Beck et al.'s [1] model is based on the health economic guidelines of their country, which differ from Dutch guidelines [4]. Based on different regulations the model by Beck et al. [1] incorporates family and network utility decrements, a quality-of-life adjustment factor, and decreased discount rates in the base case—none of which align with Dutch Health Economic Guidelines. Secondly, Beck et al. [1] applies an average incidence of 1.83/100,000 based on a 2015 report, which is much higher than the 0.66/100,000 average incidence from 2024 underpinning our model. The following pages highlight these differences in more detail.

Table 1 highlights key characteristics of our evaluation of 4CMenB in infants and the evaluations conducted by Beck et al.

|  |  | **Beck et al. (Baseline)** | **Beck et al. (Including broad disease burden categories [DBCs])** | **Our model** |
| --- | --- | --- | --- | --- |
| **Scope** | **Patient population** | Infants (age 0) | Infants (age 0) | Infants (age 0) |
|  | **Intervention** | 4CMenB | 4CMenB | 4CMenB |
|  | **Comparator** | No vaccine | No vaccine | No vaccine |
|  | **Schedule** | 2+1 schedule (2 and 4 months) | 2+1 schedule (2 and 4 months) | 2+1 schedule (2 and 4 months) |
|  | **Included costs** | Vaccine cost, administration cost, vaccine adverse event costs, acute IMD-B healthcare costs | Vaccine cost, administration cost, vaccine adverse event costs, acute IMD-B healthcare costs  DBC 1: Long term sequelae costs  DBC 3: special education, public health response costs, long term caregiving costs, litigation costs, productivity loss | Vaccine cost, administration cost, vaccine adverse event costs, acute IMD-B healthcare costs, Long term sequelae costs, special education, public health response costs, long term caregiving costs, productivity loss |
|  | **Included effects** | Acute IMD-B utility decrement, deaths due to IMD-B | Acute IMD-B utility decrement, deaths due to IMD-B  DBC 1: long term sequelae utility decrements  DBC 2: caregiver utilities, bereavement factors  DBC 4: Quality of life adjustment factor (3x) | Acute IMD-B utility decrement, deaths due to IMD-B, long term sequelae utility decrements |
|  | **Perspective** | Healthcare | DBC 3: Societal | Societal |
|  | **Time horizon** | 100 years (effectively lifetime) | 100 years (effectively lifetime) | Lifetime |
|  | **Modelling method** | Dynamic transmission model | Dynamic transmission model | State transition model |
|  | **Country** | England | England | Netherlands |
|  | **Discount rates** | 3.5% for costs and effects | DBC 5: 1.5% for costs and effects | 1.5% for costs and 3.5% for effects |
| **Key Inputs** | **Average incidence** | 1.83 | 1.83 | 0.66 |
|  | **Vaccine price** | £75 | £75 | €85.56 |
|  | **Mean duration of protection** | 38 months | 38 months | 30 months |
| **Results** | **ICER** | 360.595 £/QALY | 18.645 £/QALY | 592,279 €/QALY |
| DBC – Disease burden category | | | | |

Table 1 Key characteristics of Beck et al. analyses and our own analysis

### Differences between our model and Beck et al (Baseline)

Apart from being presented in different currencies, there are several factors contributing to the differences in results between the baseline in Beck et al. [1] and our model.

The scope of Beck and al.‘s model focuses solely on direct healthcare costs and effects, while our model takes a societal perspective, incorporating both direct and indirect costs and effects. Additionally, the analyses use different discount rates, reflecting varying health economic guidelines and contextual differences.

Key input variables also differ between the models: the incidence rate, vaccine dose price, and mean duration of protection vary between the analyses. Tables 1 and 2 demonstrate how variations in incidence and price inputs can significantly impact the ICER. Figure 1 highlights a sensitivity analysis on the duration of protection. The figures shows that the increase in duration of protection would lead to a decreased ICER. This decrease is however moderate, as IMD-B incidence is age-dependent and the prolonged duration of protection would occur in an age group in which the incidence is lower. Given the relatively small variations in price and the moderate impact of duration of protection, we think that difference in incidence is likely the most impactful difference of the three inputs.

Lastly, the modelling method differs. While Beck et al. [1] uses a dynamic transmission model, we used a state transition model. The upside of a dynamic transmission model is that the model can accommodate for indirect protection against MenB carriage acquisition caused by the vaccine. Beck et al. only applies such indirect protection dynamics in uncertainty analyses and – as our model – assumed no indirect protection in the base case analysis. While none of the presented analyses thus considers indirect protection, the differences in modelling methods and resulting model structure may still lead to differences in model results which are difficult to estimate.


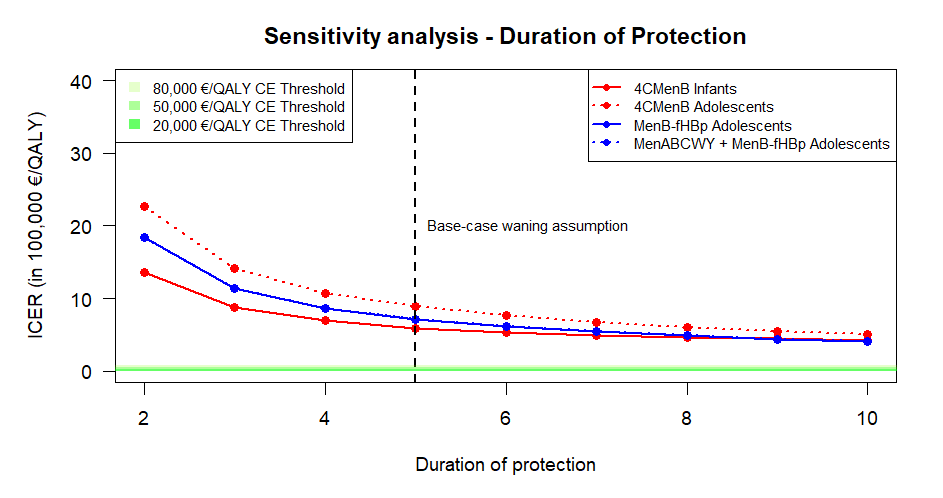


Figure 1 Sensitivity analyses of ICER to variation in duration of protection when varying the maximum duration of protection from 2 to 10 years. The green lines at the bottom indicate different cost-effectiveness thresholds. The black vertical line indicates the base-case waning input. The lines of the MenB-fHBp and MenABCWY + MenB-fHBp schedules overlap fully.

### Differences between our model and Beck et al (Including broad disease burden categories)

The second analysis adds five disease burden categories: 1.) Long term burden of sequelae, 2.) Patient spill-over effects, 3.) Societal perspective, 4.) QoL Severity modifier and 5.) adjusted discount rates. We explored a number of these disease burden categories in our scenario analyses. Figure 2 shows the results of a selection of these scenario analyses.


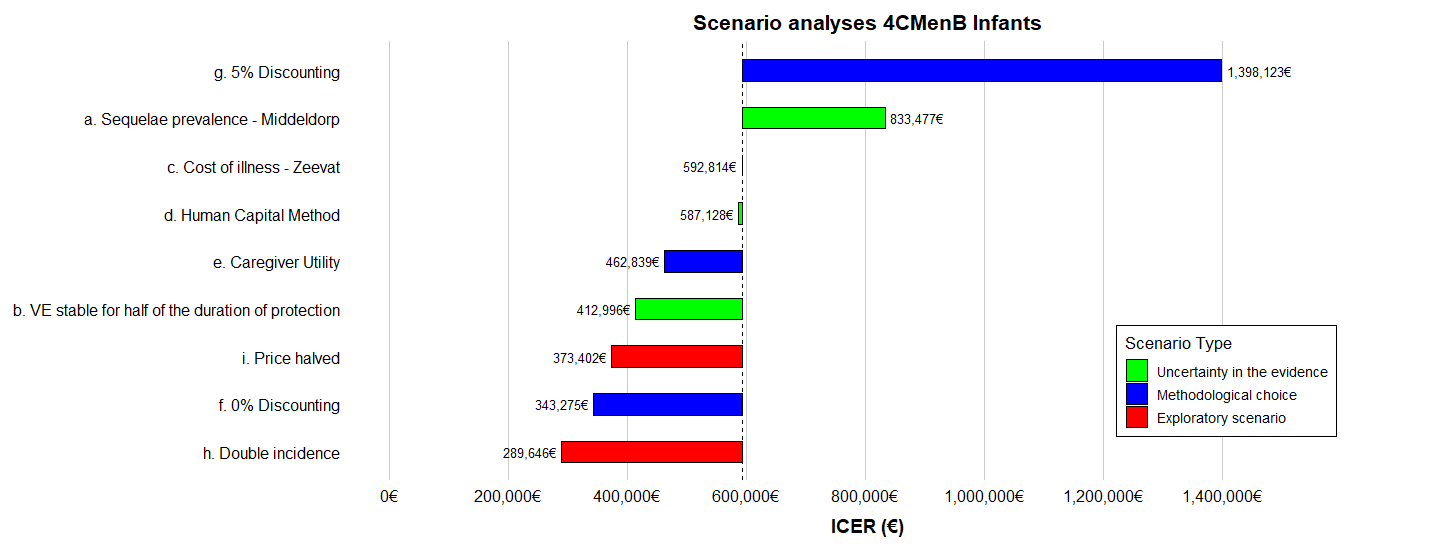


Figure 2 Results Scenario Analyses 4CMenB Infants: 3 scenario analyses about uncertainty in the evidence (a. Applying sequelae prevalence from Middeldorp[5], b. VE remains stable for half of the duration of protection, c. Applying the cost of Illness from Zeevat[6]); 4 scenario analyses about alternative methodological choices (d. Applying the Human Capital Method to value productivity, e. including caregiver utility, f. applying 0% discounting, g. applying 5% discounting); 2 Exploratory scenario analyses (h. doubling the incidence of IMD-B, i. halving the price per vaccine dose)

In 1.) Beck et al. [1] adds costs and effects of long term sequelae to their baseline model based. We included the same sequelae in our base-case analysis. This additional disease burden category should therefore not cause a substantial difference between the results of our models. Scenario analysis a. ‘Sequelae prevalence – Middeldorp’ (Figure 2) simulates the effect of applying recent Dutch evidence by Middeldorp [5] which suggests that the prevalence of sequelae decreases after one year after the occurrence of IMD-B. The analysis shows that a decreased prevalence of sequelae one year after the occurrence of IMD-B increases the ICER substantially.

In 2.) Beck et al. [1] adds quality of life decrements for the family and network of patients undergoing IMD-B. This includes quality of life decrements for caregivers, and a bereavement factor in case of patient death. Analyses e. ‘Caregiver utility’ (Figure 2) applied the same quality of life decrements with the same methodology in our analysis. The result is a decrease in the ICER from 592,279 €/QALY to 462,839 €/QALY. It should be noted that the Dutch Health Economic Guidelines [4] recommend to only use caregiver utility in scenario analyses and to limit the inclusion of caregiver quality of life to the lifetime of patients. According to Dutch Health Economic Guidelines, this analysis should only be considered as a scenario analysis and the bereavement factor should not be considered at all.

In 3.) Beck et al. [1] adds a societal perspective to their analysis. This is part of our base-case as per the Dutch Health Economic guidelines. The only difference between the included costs and effects between the analyses was that Beck et al. [1] included litigation costs which we did not include. We do not expect this to be influential.

In 4.) Beck et al. [1] adds a quality of life adjustment factor of 3 to all utility decrements in the model. This quality of life adjustment factor was based on considerations that society values the prevention of potentially devastating disease like IMD-B higher than that of relatively mild diseases. The method is in line with a decision by the UK Joint Committee on Vaccination and Immunisation. We did not conduct similar analyses, as there is no mention of this type of method in Dutch Health Economic Guidelines [4].

In 5.) Beck et al. [1] decreases discount rates for both costs and effects to 1.5%. Our exploratory scenario analysis f. ‘0% Discounting’ (Figure 2) shows the results of a similar scenario analysis which we conducted in our model. The direction of the effect is the same as in the analysis by Beck et al. [1]. The Dutch Health Economic Guidelines [4] do not recommend the use of different discount rates for technologies with a longer effect.

# TECH-VER

| **Question** | **Expected answer** | **Answer** | **Documentation** |
| --- | --- | --- | --- |
| **Does the technology (drug/device, etc.) acquisition cost increase with higher prices?** | Yes | Yes | See scenario analysis i.) |
| **Does the drug acquisition cost increase for higher weight or body surface area?** | Yes | N/A | BSA is not included |
| **Does the probability of an event, derived from an OR/RR/HR and baseline probability, increase with higher OR/RR/HR?** | Yes | Yes | See scenario analysis h.) |
| **In a partitioned survival model, does the progression-free survival curve or the time on treatment curve cross the overall survival curve?** | No | N/A |  |
| **If survival parametric distributions are used in the extrapolations or time-to-event calculations, can the formulae used for the Weibull (generalized gamma) distribution generate the values obtained from the exponential (Weibull or Gamma) distribution(s) after replacing/transforming some of the parameters?** | Yes | N/A |  |
| **Is the HR calculated from Cox proportional hazards model applied on top of the parametric distribution extrapolation found from the survival regression?** | No | N/A |  |
| **For the treatment effect inputs, if the model uses outputs from WINBUGS, are the OR, HR, and RR values all within plausible ranges? (Should all be non-negative and the average of these WINBUGS outputs should give the mean treatment effect)** | Yes | N/A |  |
| **Calculate the sum of the number of patients at each health state** | Should add up to cohort size | Yes | Included in the model as permanent check. |
| **Check if all probabilities and number of patients in a state are greater than or equal to 0** | Yes | Yes | Included as necessary part of model functionality |
| **Check if all probabilities are smaller than or equal to 1** | Yes | Yes | Included as necessary part of model functionality |
| **Compare the number of dead (or any absorbing state) patients in a**  **period with the number of dead (or any absorbing state) patients in**  **the previous periods?** | Should be larger | Yes | Included in the model as permanent check. |
| **In case of lifetime horizon, check if all patients are dead at the end**  **of the time horizon** | Yes | Yes | Checked. |
| **Set all utilities to 1** | QALYs should equal LYs | Yes | Checked. |
| **Set all utilities to 0** | QALYs should equal 0 | Yes | Checked. |
| **Decrease all state utilities simultaneously** | Lower utilities should be accumulated at all times. | Yes | Checked. |
| **Set all costs to 0** | No costs will be accumulated in the model | Yes | Checked. |
| **Put mortality rate to 0** | Patients never die | Yes | Checked. |
| **Put mortality rate at extremely high** | Patients die in the first few cycles | Yes | Checked. |
| **Set the effectiveness-, utility-, and safety-related model inputs for all**  **treatment options equal** | Same life-years and QALYs should be accumulated for all treatment at  any time | Yes | Checked. |
| **Change around the effectiveness-, utility- and safety-related model inputs between two treatment options** | Outcomes should be reversed as well | Not checked | Not possible to check in the current model setup |
| **Check if the number of alive patients estimated at any cycle is in line with general population life-table statistics** | At any given age, the percentage alive should be lower or equal in com- parison with the general population estimate | Yes | General population estimates are added to disease specific mortality |
| **Check if the QALY estimate at any cycle is in line with general population utility estimates** | At any given age, the utility assigned in the model should be lower or equal in comparison with the general population utility estimate | N/A | Population utility estimates are added to QoL estimates |
| **Set the inflation rate for the previous year higher** | The costs (which are based on a reference from previous years) assigned at  each time will be higher | Yes | Checked. |
| **Calculate the sum of all ingoing and outgoing transition probabilities of a state in a given cycle** | Difference of ingoing and outgoing probabilities at a cycle in a state times the cohort size will yield the change in the number of patients at that state in that cycle | Yes | Checked. |
| **Calculate the number of patients entering and leaving a tunnel state throughout the time horizon** | Numbers entering = numbers leaving | Yes, except for those dying of background mortality. | All patients who enter the tunnel health states, except for those who die, leave the health state after one model cycle. |
| **Check if the time conversions for probabilities were conducted correctly.** | Yes | Yes | Checked. |
| **Increase the treatment acquisition cost** | Costs accumulated at a given time will increase during the period when the treatment is administered | Yes | Checked. |
| **Set the mortality and incidence rates to 0** | Prevalence should be constant in time | Yes | Checked. |
| **Check the incremental life-years and QALYs gained results. Are they in line with the comparative clinical effectiveness evidence of the treatments involved?** | If a treatment is more effective, it generally results in positive incremental LYs and QALYs in comparison with the less-effective treatments | Yes | Vaccine is slightly more effective than no vaccine |
| **Check the incremental cost results. Are they in line with the treatment costs?** | If a treatment is more expensive, and if it does not have much effect on other costs, it generally results in positive incremental costs | Yes | Vaccine is more expensive than no vaccine |
| **Total life years greater than the total QALYs** | Yes | Yes | Checked. |
| **Undiscounted results greater than the discounted results** | Yes | Yes | Checked. |
| **Divide undiscounted total QALYs by undiscounted life years** | This value should be within the outer ranges (maximum and minimum) of all the utility value inputs | Yes (0.92) | Checked. |
| **Subgroup analysis results: How do the outcomes change if the char- acteristics of the baseline change?** | Better outcomes for better baseline health conditions, and worse outcomes for worse health conditions, are expected | N/A | The only difference in group results is for infants or adolescents. There is no clear better or wors baseline health condition. |
| **Could you generate all the results in the report from the model (including the uncertainty analysis results)?** | Yes | Yes | All the reported values resulted from the model |
| **Do the total life-years, QALYs, and costs decrease if a shorter time horizon is selected?** | Yes | Yes | Checked. |
| **Is the reporting and contextualization of the incremental results correct?** | Yes | Yes |  |
| **Are the reported ICERs in the fully incremental analysis non- decreasing?** | Yes | N/A | No fully incremental analysis was conducted |
| **If disentangled results are presented, do they sum up to the total results (e.g. different cost types sum up to the total costs estimate)?** | Yes | N/A | No disentangled results are presented |
| **Check if half-cycle correction is implemented correctly (total life- years with half-cycle correction should be lower than without)** | The half-cycle correction implementation should be error-free. Also check if it should be applied for all costs, for instance if a treatment is adminis- tered at the start of a cycle, half-cycle correction might be unnecessary | N/A | No half-cycle correction was conducted because of short cycle times |
| **Check the discounted value of costs/QALYs after 2 years** | Discounted value = undiscounted/(1 + r)2 | Yes | Checked |
| **Set discount rates to 0** | The discounted and undiscounted results should be the same | Yes | See scenario analysis f.) |
| **Set mortality rate to 0** | The undiscounted total life-years per patient should be equal to the length of the time horizon | Yes | Checked. |
| **Put the consequence of adverse event/discontinuation to 0 (0 costs and 0 mortality/utility decrements)** | The results would be the same as the results when the AE rate is set to 0 | N/A | Adverse events are only included as costs |
| **Divide total undiscounted treatment acquisition costs by the average duration on treatment** | This should be similar to treatment-related unit acquisition costs | Yes | Checked. |
| **Set discount rates to a higher value** | Total discounted results should decrease | Yes | See scenario analysis g.) |
| **Set discount rates of costs/effects to an extremely high value** | Total discounted results should be more or less the same as the discounted  results accrued in the first cycles | Yes | Checked. |
| **Put adverse event/discontinuation rates to 0 and then to an extremely high level** | Less costs and higher QALYS/LYs when adverse event rates are 0, higher costs and lower QALYS/LYs when AE rates are extreme | Yes | Checked. |
| **Double the difference in efficacy and safety between the new intervention and comparator, and report the incremental results** | Approximately twice the incremental effect results of the base case. If this is not the case, report and explain the underlying reason/mechanism | N/A | The translation of efficacy to cost-effectiveness does not work as straight forward here as in other drugs. |
| **Do the same for a scenario in which the difference in efficacy and safety is halved** | Approximately halve of the incremental effect results of the base case. If this is not the case, report and explain the underlying reason/mechanism | N/A | The translation of efficacy to cost-effectiveness does not work as straight forward here as in other drugs. |
| **Are all necessary parameters subject to uncertainty included in the OWSA?** | Yes | Yes | Checked. |
| **Check if the OWSA includes any parameters associated with joint uncertainty (e.g. parts of a utility regression equation, survival curves with multiple parameters)** | No | No | Checked. |
| **Are the upper and lower bounds used in the one-way sensitivity analysis using confidence intervals based on the statistical distribution assumed for that parameter?** | Yes | No | Checked. |
| **Are the resulting ICER, incremental costs/QALYs with upper and lower bound of a parameter plausible and in line with a priori expectations?** | Yes | Yes | Checked. |
| **Check that all parameters used in the sensitivity analysis have appropriate associated distributions – upper and lower bounds should surround the deterministic value (i.e. upper bound ≥ mean ≥ lower bound)** | Yes | Yes | Checked. |
| **Standard error and not standard deviation used in sampling** | Yes | Yes | Checked. |
| **Lognormal/gamma distribution for HRs and costs/resource use** | Yes | N/A | No HRs |
| **Beta for utilities and proportions/probabilities** | Yes | Yes | Checked. |
| **Dirichlet for multinomial** | Yes | N/A |  |
| **Multivariate normal for correlated inputs (e.g. survival curve or regression parameters)** | Yes | N/A |  |
| **Normal for other variables as long as samples do not violate the requirement to remain positive when appropriate** | Yes | Yes | Checked. |
| **Check PSA output mean costs, QALYs, and ICER compared with the deterministic results. Is there a large discrepancy** | No | No | Checked. |
| **If you take new PSA runs from the Microsoft Excel model do you**  **get similar results?** | Yes | Yes | Checked. |
| **Check the correlation between two PSA results (i.e. costs/QALYs**  **under the SoC and costs/QALYs under the comparator)** | Should be very low | Not very low: Correlation around 0.7 | Checked. |
| **If a certain seed is used for random number generation (or previously**  **generated random numbers are used), check if they are scattered**  **evenly between 0 and 1 when they are plotted** | Yes | N/A | No seed used. |
| **Compare the mean of the parameter samples generated by the model**  **against the point estimate for that parameter; use graphical methods**  **to examine distributions, functions** | The sample means and the point estimates will overlap, the graphs will be  similar to the corresponding distribution functions (e.g. normal, gamma,  etc.) | Yes. Example – case-fatality rate for IMD-B patients over the age of 65. | 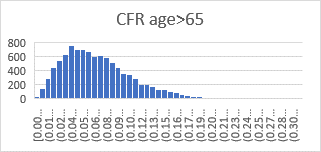 |
| **Check if sensitivity analyses include any parameters associated with**  **methodological/structural uncertainty (e.g. annual discount rates,**  **time horizon)** | No | Yes | Structural parameters are included in the scenario analyses. |
| **Value of information analysis if applicable: Was this implemented**  **correctly?** | Yes | N/A | No VOI analyses conducted |
| **Which types of analysis? Were aggregated parameters used? Which**  **parameters are grouped together? Does it match the write-up’s**  **suggestions?** | Yes | N/A | No VOI analyses conducted |
| **Is EVPI larger than all individual EVPPIs?** | Yes | N/A | No VOI analyses conducted |
| **Is EVPPI for a (group of) parameters larger than the EVSI of that**  **(group) of parameter(s)?** | Yes | N/A | No VOI analyses conducted |
| **Are the results from EVPPI in line with OWSA or other parameter**  **importance analysis (e.g. ANCOVA)?** | Yes | N/A | No VOI analyses conducted |
| **Did the electronic model pass the black-box tests of the previous**  **verification stages in all PSA iterations and in all scenario analysis**  **settings?** | Yes | No | The verification was only conducted for the deterministic base-case. |
| **Check if all sampled input parameters in the PSA are correctly**  **linked to the corresponding event/state calculations** | Yes | Yes | Sampled input parameters are recorded through their link to the calculations. |
